# A serine/threonine protein kinase encoding gene *KERNEL NUMBER PER ROW6* regulates maize grain yield

**DOI:** 10.1038/s41467-020-14746-7

**Published:** 2020-02-20

**Authors:** Haitao Jia, Manfei Li, Weiya Li, Lei Liu, Yinan Jian, Zhixing Yang, Xiaomeng Shen, Qiang Ning, Yanfang Du, Ran Zhao, David Jackson, Xiaohong Yang, Zuxin Zhang

**Affiliations:** 1 National Key Laboratory of Crop Genetic Improvement, College of Plant Science and Technology, Huazhong Agricultural University, Wuhan, 430070 P. R. China; 2 State Key Laboratory of Plant Physiology and Biochemistry, National Maize Improvement Center of China, MOA Key Lab of Maize Biology, Beijing Key Lab of Crop Genetic Improvement, China Agricultural University, Beijing, 100193 P. R. China; 3 Cold Spring Harbor Laboratory, Cold Spring Harbor, New York, NY 11724 USA

**Keywords:** Agricultural genetics, Agricultural genetics, Plant breeding, Transgenic plants

## Abstract

Increasing grain yield of maize (*Zea mays* L.) is required to meet the rapidly expanding demands for maize-derived food, feed, and fuel. Breeders have enhanced grain productivity of maize hybrids by pyramiding desirable characteristics for larger ears. However, loci selected for improving grain productivity remain largely unclear. Here, we show that a serine/threonine protein kinase encoding gene *KERNEL NUMBER PER ROW6 (KNR6)* determines pistillate floret number and ear length. Overexpression of *KNR6* or introgression of alleles lacking the insertions of two transposable elements in the regulatory region of *KNR6* can significantly enhance grain yield. Further in vitro evidences indicate that KNR6 can interact with an Arf GTPase-activating protein (AGAP) and its phosphorylation by KNR6 may affect ear length and kernel number. This finding provides knowledge basis to enhance maize hybrids grain yield.

## Abstract (web summary)

Selection of kernel number per ear has improved maize yield, but the genetic base is unclear. Here, the authors reveal that a serine/threonine protein kinase KNR6 is a positive regulator of the trait and show *in vitro* evidences that KNR6 may function through phosphorylating an Arf GTPase-activating protein.

## Introduction

Maize (*Zea mays* L.) is an economically important and globally cultivated crop. Increasing maize grain yield has long been a key target in maize breeding. The kernel number per row (KNR) of maize is a key trait that contributes greatly to grain yield per ear. KNR is associated with the number of pistillate florets that are generated during inflorescence development, as well as floret fertility. A greater number of florets and higher floret fertility provide a means for developing more kernels per ear. During ear inflorescence development, reproductive axillary meristems develop into pistillate florets. Analyses of mutants have established that the *PINOID*-related kinase gene *BARREN INFLORESCENCE2* (*BIF2*)^1,2^ and the two *AUXIN/INDOLE-3-ACETIC ACID* (*AUX/IAA*)-related genes *BIF1* and *BIF4*^3^ function in inflorescence axillary meristem initiation and determinacy. In addition, two auxin biosynthesis genes, *SPARSE INFLORESCENCE* (*SPI1*)^4^ and *VANISHING TASSEL2* (*VT2*)^5^, are required for inflorescence development. These results indicate that auxin plays a critical role in regulation of the number of florets on the maize ear. Genetic analyses have identified a set of quantitative trait loci (QTLs) controlling KNR variation^6^. Characterization of these KNR QTLs may provide genetic and molecular knowledge of inflorescence development that can enhance breeding efforts for improving grain yield^7–10^. Nonetheless, no natural causal variants associated with KNR have been elucidated.

Transposable elements (TEs) or transposons are mobile genetic units that were first discovered in maize^11^, and are universally present in living organisms. By moving within a genome, TEs alter gene regulation and genome complexity, and genetic diversity created by TEs is a key source of functional variation with profound significance^12–15^. The maize genome is highly complex and harbors many types of TEs that account for ~85% of the genome^16–19^. TEs contribute to the wide genetic diversity among both wild and cultivated relatives^20,21^, producing variability in domestication, improvement, and adaptation traits^22–24^. For example, a *Hopscotch* TE insertion in the regulatory region of *TEOSINTE BRANCHED1* (*TB1*) creates an enhancer that activates *TB1* expression to increase apical dominance and repress axillary bud outgrowth^22^. Similarly, a *Harbinger*-like TE insertion 57 kb upstream of *ZmCCT9* and a CACTA-like TE insertion in the *ZmCCT10* promoter have created new flowering time variants targeted by selection to allow maize spread from its tropical origin to higher latitudes^23,24^.

In this study, we clone the *qKNR6* QTL, and find that it encodes a serine/threonine protein kinase that regulates KNR through control of floret number and ear length (EL). Two TE presence/absence variation (PAV) polymorphisms in the regulatory region of *KNR6* are major variants, with strong effects on KNR, EL, and grain yield. We also show a regulatory pathway of *KNR6* on the ear development and grain yield in maize.

## Results and discussion

### Positional cloning of *qKNR6*

**Fig. 1:**
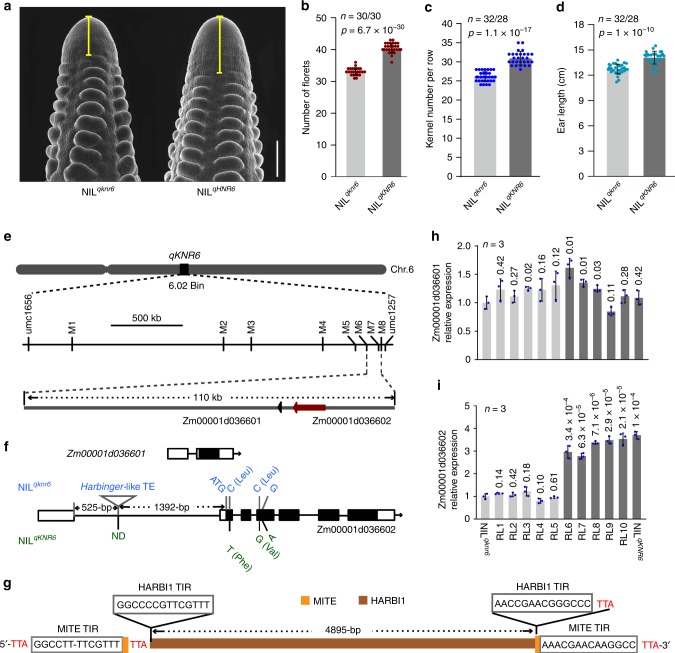
Phenotype of two QTL parental lines and map-based cloning of *qKNR6*. **a** Ear inflorescence meristems of the two parents. Scale bar = 500 μm. **b**–**d** Comparisons of floret number (**b**), EL (**c**), and KNR (**d**) between NIL^*qknr6*^ and NIL^*qKNR6*^ grown in the field at Sanya, China in 2015. The values in (**b**–**d**) are shown as the means ± s.d., and significance was estimated by the one-way ANOVA. The numeral on the bottom of each column is the number of ears examined. **e** Fine mapping of *qKNR6. qKNR6* was located on chromosome 6, bin02. The refined 110_kb region at the *qKNR6* locus contained two genes, *Zm00001d036601* and *Zm00001d036602*. **f** Gene structure of two candidate genes and polymorphisms between the two parents. No polymorphism is identified in *Zm00001d036601*, but three SNPs in exons and a Harbinger-like TE (triangle) in the 5′-UTR intron of *Zm00001d036602* were detected. **g** A structural diagram of the TE inserted in *KNR6*, predicted using CENSOR. **h**, **i** Expression of *Zm00001d036601* (**h**) and *Zm00001d036602* (**i**) in the Ims of ten recombinant lines and two parental lines. Gene-expression level is analyzed using quantitative PCR with three biological replicates, each with three technical replicates. The maize *Actin* gene (*Zm00001d010159*) is used as an internal control. The values in (h and i) are shown as the means ± s.d., and *p* value is estimated by the Duncan’s test. Source data underlying Fig. 1b–d are provided in a Source Data file.

*qKNR6* was previously mapped on chromosome 6 using an F_2_ population derived from the crossing of an elite inbred line Ye478 (referred to as NIL^*qknr6*^) to SL57, which is a near-isogenic line under Ye478 genetic background (referred to as NIL^*qKNR6*^), and produces greater KNR than does NIL^*qknr6*^^25^. Analysis of NIL^*qKNR6*^ harboring the desirable *qKNR6* allele indicated that this QTL had pleiotropic effects on ear-related traits, without changes in plant architecture (Supplementary Fig. 1a–d, Supplementary Table 1). NIL^*qKNR6*^ plants had longer inflorescence meristems (IMs) (Fig. 1a) and generated more florets per row (40.5 ± 1.48) than NIL^*qknr6*^ plants (33.1 ± 1.07) on 2-cm ear primordia (Fig. 1b), suggesting that the IM of NIL^*qKNR6*^ plants had a stronger ability to produce florets. After pollination, ~76.6% of the NIL^*qKNR6*^ ear florets developed into kernels, similar to the value for NIL^*qknr6*^ florets (~79.2%). Therefore, the additional florets generated by the NIL^*qKNR6*^ plants resulted in longer ears, with KNR increasing by 17.5% and EL by 8.2% (Fig. 1c, d). Therefore, *qKNR6* affects ear traits by regulating floret production by the ear inflorescence meristem.

To identify *qKNR6*, we backcrossed NIL^*qKNR6*^ to NIL^*qknr6*^ and obtained ten recombinant chromosomes from a selfed-backcross population of ~28,000 individuals. Using recombinant-derived progeny testing, we delineated *qKNR6* to an ~110_kb interval flanked by markers M6 and M8 (Fig. 1e). Homozygous recombinants harboring *qKNR6* alleles within the M6–M8 interval showed an increase in KNR, EL, and ear weight (EW), but no change in KRN or ED, as expected, in two planting seasons (Supplementary Fig. 1e, f; Supplementary Fig. 2a–d). We found two predicted genes, *Zm00001d036601* and *Zm00001d036602*, within the 110_kb interval (B73 RefGenV4, Fig. 1e). Although there were no differences between the QTL parents in the coding region of *Zm00001d036601*, we found four polymorphic sites in *Zm00001d036602*, including a 5054_bp TE-PAV in the intron within the 5′-UTR, and three single-nucleotide polymorphisms (SNPs) in exons. Two of these SNPs were nonsynonymous, including a C/T transition that leads to replacement of a leucine with a phenylalanine (L_7_F) and a C/G transversion that leads to replacement of a leucine with a valine (L_70_V) (Fig. 1f). The 5054_bp TE consisted of two elements: a 126_bp *MITE* (miniature inverted-repeat transposable element) flanked by a direct repeat of the TTA trinucleotide that was interrupted by insertion of a larger element of 4926_bp (Fig. 1g). This larger element possessed a HARBI1-putative nuclease-encoding sequence, and 14_bp terminal-inverted repeat (TIR) flanking sequences and a 3_bp (TTA) direct repeat, with typical features of a *Harbinger* TE^26,27^ (Fig. 1g). Hereafter, we refer to the 5054_bp sequence as a *Harbinger*-like TE. In addition to these obvious polymorphisms, *Zm00001d036602* was differentially expressed in IMs between the QTL parents, whereas the adjacent gene *Zm00001d036601* was not (Fig. 1h, i). Significantly, KNR and EL traits in the recombinants correlated highly with expression of *Zm00001d036602* (*r* = 0.97 and 0.92, *p* = 1.18 × 10^−7^ and 2.50 × 10^−5^, the two-tailed Student’s *t* test, respectively; Supplementary Fig. 3a, b) but not with *Zm00001d036601* (Supplementary Fig. 3c, d). We therefore propose *Zm00001d036602* as the candidate gene for *qKNR6* and hereafter refer to it as *KNR6*.

Rapid amplification of cDNA ends (RACE)-polymerase chain reaction (PCR) revealed that the two QTL parents produced transcripts with identical length (1872 bp), and they encoded proteins of 381 amino acids containing a conserved serine/threonine-protein kinase domain (Supplementary Fig. 4a–d; Supplementary Fig. 5a). The seventh amino acid (L) of KNR6 was polymorphic between QTL parents and among common wheat, indica rice, and barley, although it was conserved in japonica rice (Os06T0676600), Sorghum (SORBI_010G228000) and Brachypodium (BRADI1G32630). The 70th amino acid (L) was not conserved across grass species (Supplementary Fig. 5b, c).

### *KNR6* is the causal gene underlying *qKNR6*

**Fig. 2:**
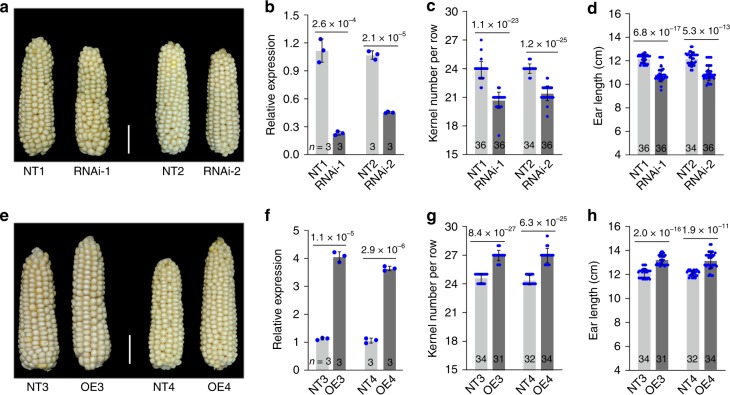
Function of *KNR6* on kernel number per row and ear length. **a** *KNR6* RNAi transgenic lines (RNAi-1 and RNAi-2) have shorter ears than do non-transgenic sib lines (NTs). **b**, **f** *KNR6* expression in two RNAi lines (**b**) and two *KNR6*-overexpressing lines (**f**). Gene expression level is analyzed using quantitative PCR with three biological replicates, and each with three technical replicates. The maize *Actin* gene (*Zm00001d010159*) is used as an internal control. *p* Value is estimated using the two-tailed Student’s *t* test. **c**, **d** Phenotypes of KNR (**c**) and EL (**d**) in two RNAi lines. **e** *KNR6*-overexpressing lines (OE3 and OE4) have longer ears than do non-transgenic sib lines (NTs). **g**, **h** Phenotypes of KNR (**g**) and EL (**h**) in two *KNR6*-overexpressing lines. The values in (**c**, **d**, **g**, **h**) are means ± s.d. Number on the bottom of each column is the sample size. Significance is estimated by the one-way ANOVA. Scale bar = 3 cm in (**a**) and (**e**). Source data underlying Fig. 2c, d are provided in a Source Data file.

To evaluate the candidate gene, we generated transgenic maize lines with either silencing of the *KNR6* candidate via RNA interference (*KNR6*-RNAi) or with *KNR6* overexpression (*KNR6*-OE) in inbred line A188. Two independent *RNAi* transgenic lines (RNAi-1 and RNAi-2) and two overexpressing lines (OE3 and OE4) were analyzed in the T_3_ generation (Fig. 2a–h, Supplementary Table 2). Compared with the respective non-transgenic (NT) lines, the RNAi lines had shorter ears (by 1.35 ± 0.11 cm, *p* = 2.05 × 10^−14^, the two-tailed Student’s *t* test) as expected, with fewer kernels per row (by 2.85 ± 0.22 KNR, *p* = 1.37 × 10^−25^, the two-tailed Student’s *t* test) (Fig. 2a, c, d). In contrast, the overexpressing lines produced longer ears (by 1.1 ± 0.26 cm, *p* = 3.53 × 10^−15^, the two-tailed Student’s *t* test) with more kernels per row (by 2.5 ± 0.15 KNR, *p* = 1.85 × 10^−28^, the two-tailed Student’s *t* test) (Fig. 2e, g, h). These phenotypic changes corresponded with the expression levels of *KNR6* in the transgenic lines (Fig. 2b, f), indicating that expression of *KNR6* correlated positively with KNR, consistent with *KNR6* expression and phenotypic performance in NIL^*qKNR6*^ and NIL^*qknr6*^. These data strongly support that *Zm00001d036602* is the causal gene underlying *qKNR6*, and changing its expression controls KNR variation.

### Two PAVs in qKNR6 are associated with KNR

**Fig. 3:**
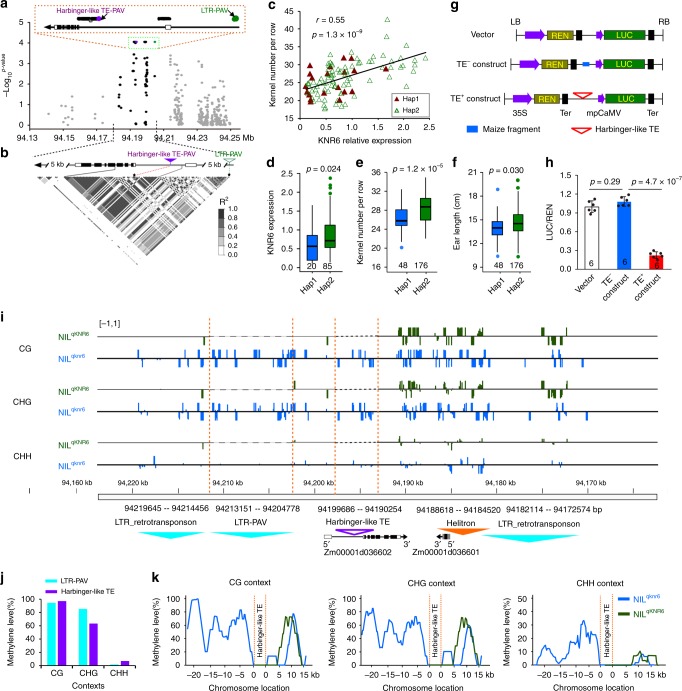
Association mapping and DNA methylation assay. **a** Associations of 433 DNA polymorphisms at the *KNR6* locus with KNR in 224 diverse maize inbred lines. Each dot represents a polymorphic site. Black dots, polymorphic sites within 10_kb flanking sequences of *KNR6*; purple dot, the *Harbinger*-like TE PAV; green dot, the LTR-TE PAV; gray dots, others. Black rectangles, exons; white rectangles, UTRs. The significance level of the associated site, *p* = 0.05/the number of polymorphic sites. **b** The pattern of linkage disequilibrium for polymorphic sites. Asterisks, two PAV sites in the LD block. **c** Pearson correlation between *KNR6* expression levels and KNR. The *p* value is determined by the two-tailed Student’s *t* test. **d** Expression levels of *KNR6* in 20 *Hap1* lines and 85 *Hap2* lines using qPCR, with three biological replicates in each case. **e**, **f** Box-and-whisker plots of KNR (**e**) and EL (**f**) for 48 *Hap1* lines and 176 *Hap2* lines. Each box represents the median and interquartile range. Whiskers extend to maximum and minimum values. Significance of difference is estimated by the one-way ANOVA. **g** A diagram of the constructs. *TE*^*+*^ construct includes a 5570_bp fragment containing the *Harbinger*-like TE; *TE*^*−*^ construct represents the 567_bp sequence lacking the *TE*. **h** Luciferase activity in maize leaf protoplasts. Luciferase activity is measured with three biological replicates, each with two technical replicates. Data are normalized with respect to the average value of the empty construct, and are shown as means ± s.d. *p* Value is estimated by the one-way ANOVA. **i** Visualization of the DNA methylation regions. DNA methylation levels were measured by bisulfite sequencing, and the methylation status was illustrated using the Integrated Genome Viewer. The upper and lower panels show the sense and antisense strands, respectively. **j** DNA methylation level of the three contexts in the long terminal repeat (LTR) retrotransposon and the *Harbinger*-like TE. **k** Methylation patterns of the flanking regions of the *Harbinger*-like TE in both parent lines. The methylation levels of CG, CHG, and CHH were scanned using a window size of 200 bp and a step size of 100 bp. Source data underlying Fig. 3e–h are provided in a Source Data file.

We next sequenced *KNR6* in a set of 224 diverse maize inbred lines. A long terminal repeat (LTR) retrotransposon PAV (referred to as LTR-PAV) was identified in the promoter, ~5.1_kb upstream of the *KNR6* transcription start site, in the association panel and between the two parental lines. In addition, a total of 433 variants with minor allele frequency ≥0.05 were detected within the 100_kb region centered on *KNR6*. Of these, 52 variants, including the TE-PAV in the 5ʹ-UTR intron, and the LTR-PAV in the promoter, were significantly associated with KNR (−log_10_(*P*) > 3.94) (Fig. 3a, Supplementary Data 1). These KNR-associated variants were located in a strong linkage disequilibrium (LD) block (Fig. 3b, Supplementary Data 1). In addition, *KNR6* expression levels in ~1.5–2.0-mm immature ears of 105 inbred lines also correlated positively with KNR (*r* = 0.55, *p* = 1.33 × 10^−9^, the two-tailed Student’s *t* test) (Fig. 3c). Because both the TE-PAV and LTR-PAV were located in regions with the potential to regulate gene expression, two markers were developed and used to identify haplotypes in the association panel: haplotype 1 (*Hap1*) including 48 inbred lines with the TE-PAV and the LTR-PAV, and haplotype 2 (*Hap2*) including 176 inbred lines lacking the transposons. The average expression level of *KNR6* in the *Hap2* lines was significantly higher than that in the *Hap1* lines (Fig. 3d), and the *Hap2* lines had longer ears with greater KNR than the *Hap1* lines (Fig. 3e, f). These effects agree with the phenotypic changes observed in the NIL overexpression and RNAi lines, suggesting that the two PAVs in *qKNR6* contribute to the phenotypic variation in KNR and EL across a wide range of maize germplasm.

Next, to explore the effect of the 5ʹ-UTR *Harbinger*-like TE on gene expression, we amplified two types of 5′-UTR intron segments, including a 567_bp fragment lacking the TE, and a 5570_bp fragment containing it (Supplementary Fig. 6). We cloned each fragment upstream of a luciferase (LUC) reporter construct driven by the cauliflower mosaic virus 35S minimal promoter (mpCaMV) (Fig. 3g), and compared LUC activity in maize leaf protoplasts (Fig. 3h). The results showed significantly lower *LUC* activity in the construct having the *Harbinger*-like TE (*TE*^*+*^ construct) relative to the construct lacking it (*TE*^*−*^ construct), while activity in the *TE*^*−*^ construct was not significantly different from that in the control empty vector (Fig. 3h). These results suggest that the *Harbinger*-like TE represses gene expression, and we infer that it likely functions to reduce *KNR6* expression in vivo.

TE insertions can influence nearby gene expression by inducing DNA methylation of flanking sequences^28–30^. Thus, to determine whether the *Harbinger*-like TE and the LTR retrotransposon participated in the epigenetic regulation of *KNR6* expression, we measured DNA methylation levels in an ~30_kb genomic region centered on *KNR6* in both QTL parents by bisulfite sequencing (Fig. 3i). In NIL^*qknr6*^, we found that both the LTR-PAV and the TE-PAV were hypermethylated in CG (94.5 and 97.1%) and CHG (85.2 and 63.4%) contexts but not in CHH (1.7 and 7.0%) (Fig. 3j). Moreover, the average methylation level in the 5–15_kb region upstream from the *Harbinger*-like TE was dramatically higher in NIL^*qknr6*^ than in NIL^*qKNR6*^ (Fig. 3I, k), while the regions immediately flanking the *Harbinger*-like TE-PAV demonstrated low methylation (Fig. 3i, k). This result agreed with the observation that genic transposons do not always spread methylation^30^. The heavily methylated regions 5–15_kb upstream from the *Harbinger*-like TE include the LTR-PAV, suggesting that methylation differences in the upstream region of *KNR6* between the two parental lines may be caused by the LTR-PAV 5.1_kb upstream of *KNR6*, not by the *Harbinger*-like TE-PAV within the first intron of *KNR6*. In addition, a high methylation level was found in both parents within the 5_kb region downstream of *KNR6*, and was associated with a *Helitron* TE and a LTR retrotransposon (Fig. 3i–j). We confirmed the difference in methylation levels by *Hpa* I, *Msp* I, and *Alu* I digestion of the regions flanking the Harbinger-like TE, followed by quantitative PCR (Supplementary Fig. 7a–d), and found elevated DNA methylation in the upstream region of *KNR6* in NIL^*qknr6*^. This hypermethylation was also confirmed in three genetic backgrounds, B73, Zheng58, and HZ4 (Huangzao 4), having the *Harbinger*-like TE in the 5′-UTR intron and the LTR retrotransposon upstream of *KNR6*, where three sequence contexts (CG, CHG, and CHH) showed a high methylation level (Supplementary Fig. 7e), while DNA methylation could not be detected in lines lacking the harbinger-like TE in *KNR6*.

### KNR6 may phosphorylate an Arf GTPase-activating protein

**Fig. 4:**
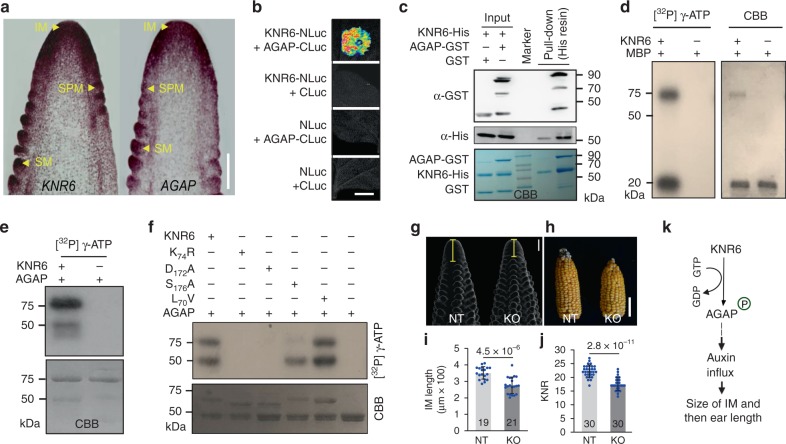
KNR6-interacting proteins and phenotypes of AGAP-knockout lines. **a** mRNA in situ hybridization with antisense probes of *KNR6* (left) and AGAP (right). Arrows point out the inflorescence meristem (IM), spikelet- paired meristem (SPM), and spikelet meristem (SM), respectively. **b**, **c** Confirmation of the KNR6-AGAP interaction by firefly luciferase complementation imaging assay (**b**) and pull-down assay (**c**). Recombinant KNR6-His protein was incubated with GST or GST-tagged AGAP, and then was bound to His resin, respectively. The eluates were resolved by SDS-PAGE and blotted using anti-GST and anti-His antibodies. MW, molecular weight. **d**, **e** Protein kinase activity of KNR6 and mutated proteins. KNR6 phosphorylated itself and myelin basic protein (MBP) (**d**) and AGAP (**e**) in vitro. **f** The kinase activity of mutated KNR6 proteins. The phosphorylation reaction was carried out using [^32^P] γ-ATP, and the phosphorylated proteins were detected by autoradiography. CBB Coomassie brilliant blue, K_74_R lysine 74 is substituted by arginine, D_172_A asparagine 172 is substituted by alanine, S_176_A serine 176 is substituted by alanine, and L_70_V leucine 70 is substituted by valine. **g**, **h** Ear inflorescences (**g**) and ears (**h**) of the AGAP-knockout line and its non-transgenic sib line. **i**, **j** The length of the inflorescence meristem (**i**) and kernel number per row (**j**) are reduced in the AGAP-knockout line compared with its non-transgenic sib line (NT). The numeral on the bottom of each column is the number of inflorescences in (**i**), and the number of ears examined in (**j**). **k** A model to illustrate the regulatory pathways of KNR6 in the ear inflorescence. KNR6 may function in auxin-dependent inflorescence development by mediating AGAP phosphorylation. A plus sign (+) means that a given substrate has been added; a minus sign (−) indicates that a given substrate has not been added. Scale bars = 100 μm in (**a**), 1 cm in (**b**), 200 μm in (**g**), and 5 cm in (**h**). Source data underlying Fig. 4c–f, i, j are provided in a Source Data file.

*KNR6* was constitutively expressed in roots, internodes, seedling leaves, mature leaves, immature ears, and tassels (Supplementary Fig. 8a), and highly enriched in inflorescence meristem (IM) and spikelet-paired meristems (SPMs) (Fig. 4a, Supplementary Fig. 8b). An in vivo immunoprecipitation assay using an anti-KNR6 antibody identified 58 KNR6-interacting proteins (Supplementary Data 2), including an Arf GTPase-activating protein (AGAP) and two 14-3-3 proteins. We verified the KNR6-AGAP interaction using firefly LUC complementation and pull-down assays (Fig. 4b, c). KNR6 and AGAP were co-expressed, supporting the likelihood of their interaction (Fig. 4a, Supplementary Fig. 8b, c). In support of the significance of their interaction, we detected KNR6 phosphorylation activity on AGAP, and on MBP (myelin basic protein), as well as KNR6 autophosphorylation activity (Fig. 4d, e). Mutation of the KNR6 protein by substitution at the 70th amino acid (L_70_V), a missense variation identified in the QTL parents, did not impact its autophosphorylation or phosphorylation activity. However, substitutions at the ATP-binding site (K_74_R) or the kinase-active site (D_172_A) resulted in loss of both autophosphorylation and phosphorylation activities (Fig. 4f). Furthermore, mutation at the 176th amino acid (S_176_A: serine replaced by alanine) severely attenuated its phosphorylation activity (Fig. 4f), suggesting that KNR6 functions as a kinase to mediate AGAP phosphorylation. To confirm the possible role of AGAP in KNR6-mediated ear development, we generated a loss-of-function line by CRISPR/Cas9 (Supplementary Fig. 9). Consistent with our model, mutation of *AGAP* resulted in a shorter IM (by ~76 μm) (Fig. 4g, i) and fewer kernels per row (by ~4.75 KNR) (Fig. 4h, j) than NT siblings. AGAP family proteins inactivate the GTPase activity of ADP ribosylation factor (Arf) small G proteins, by inducing hydrolysis of Arf-GTP to Arf-GDP^31^. G protein-coupled receptor kinases comprise a variety of serine/threonine protein kinases that interact and phosphorylate G protein-coupled receptors and AGAP^32,33^. For instance, rice OsAGAP and Arabidopsis GNOM ARF GEF regulate the localization and transport of auxin, to control auxin-dependent root development^34–38^. In maize inflorescence development, auxin biosynthesis, transport, and signaling directly control the initiation and formation of axillary meristems^1–5,39–43^. Therefore, we propose that KNR6 functions in auxin-dependent inflorescence development, by mediating AGAP phosphorylation (Fig. 4k).

The closest orthologs of *KNR6* in *Sorghum bicolor* and *Oryza sativa* have not been functionally characterized, though a close homolog in *Arabidopsis thaliana* has been found to function in response to cold stress by mediating phosphorylation of 14-3-3 proteins^44^. We also identified two 14-3-3 proteins that interacted with KNR6 (Supplementary Fig. 10), and were phosphorylated by KNR6. However, it is unknown whether KNR6 also functions in inflorescences via 14-3-3 protein phosphorylation.

### The haplotype 2 allele of KNR6 enhances hybrid grain yield

**Fig. 5:**
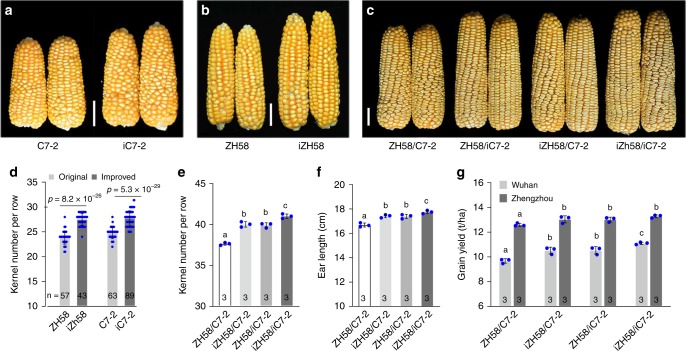
Genetic effect of *KNR6 Haplotype2* on maize grain yield. **a** Ears of inbred line Chang7-2 and its improved line Chang7-2^*qKNR6*^. **b** Ears of the inbred line Zheng58 and its improved line Zheng58^*qKNR6*^. **c** Ears of hybrids Zheng58 × Chang7-2, Zheng58 × Chang7-2^*qKNR6*^, Zheng58^*qKNR6*^ × Chang7-2, and Zheng58^*qKNR6*^ × Chang7-2^*qKNR6*^. **d** Kernel number per row in the original and improved lines. Both Zheng58 and Chang7-2 were improved by introducing the *qKNR6* desirable allele using marker-assisted backcrossing. The values are means ± s.d.; *p* value is estimated by the one-way ANOVA. **e**–**g** Phenotypic evaluation of KNR (**e**), EL (**f**), and grain yield (**g**) in hybrids carrying one or two desirable alleles. Phenotypic evaluations of the original hybrid Zheng58/Chang7-2 and improved hybrid Zheng58^*qKNR6*^/Chang7-2^*qKNR6*^, were performed at Wuhan (30.60°N, 114.30°E) and Zhengzhou (ZZ, 34.75°N, 113.62°E) in 2017 spring with randomized block design, respectively. Different letters in (**e**, **f**, **g**) at the top of each column indicate a significant difference at *p* < 0.05 determined by the Tukey HSD test. *n* is the number of ears examined in (**d**) and the number of blocks in (**e**, **f**, **g**). C7-2, Chang7-2; iC7-2, improved line Chang7-2^*qKNR6*^; ZH58, Zheng58; iZH58, improved line Zheng58^*qKNR6*^. Scale bars = 2 cm in (**a**–**c**). Source data underlying Fig. 5d–g are provided in a Source Data file.

To further verify the association between haplotype 2 allele (*Hap2*) and KNR and EL, we backcrossed the *qKNR6 Hap2* allele into two *Hap1* inbred lines, Zheng58 and Chang7-2, which are both widely used in Chinese maize-breeding programs. When the *Hap1* allele was substituted by *Hap2*, the two lines showed an increase in KNR and EL relative to the original lines (Fig. 5a, b, d; Supplementary Table 3). Notably, as a result of the increase in KNR and EL, the grain yield of the improved hybrids was significantly higher than that of the original Zheng58/Chang7-2 hybrid (13.3 ton/ha compared with 12.6 ton/ha, 5.6% increase, in Zhengzhou, and 11.0 ton/ha compared with 9.7 ton/ha, 13.4% increase, in Wuhan) (Fig. 5c, e–g). We also crossed 21 additional lines, including 11 *Hap1* lines and 10 *Hap2* lines to both NIL^*qknr6*^ and NIL^*qKNR6*^, respectively. Compared with hybrids resulting from crosses with NIL^*qknr6*^, the EL of hybrids crossed from NIL^*qKNR6*^ increased by 4.6–13.7%, and KNR increased by 4.3–19.1% under diverse genetic backgrounds (Supplementary Fig. 11a). The NIL^*qKNR6*^ hybrids with the *Hap2*/*Hap2* genotype had an average KNR increase of ~10 kernels compared with the NIL^*qknr6*^ hybrids with the *Hap1/Hap1* genotype (46.53 ± 1.87 compared with 36.44 ± 1.71 KNR, *p* = 6.32 × 10^−8^), and *Hap2*/*Hap1* heterozygous hybrids showed intermediate values (41.48 ± 2.05 and 41.96 ± 5.19 KNR, respectively) (Supplementary Fig. 11b). On average, introduction of one *Hap2* allele increased KNR by ~5.30 and EL by 1.46 cm in hybrids. Moreover, the effect of the *Hap2* allele on increasing KNR and EL was associated with higher *KNR6* expression compared with the *Hap1* lines (*p* = 0.017, the two-tailed Student’s *t* test) (Supplementary Fig. 11c). Therefore, the *Hap2* allele of *qKNR6* is desirable for increasing KNR in both inbred lines and hybrids, and for increasing hybrid EW. Similar improvements were also observed when crossing nine different inbred lines to the *KNR6*-overexpressing line (Supplementary Fig. 12a–e). These results confirm the value of *KNR6* for genetic improvement of hybrid maize kernel number, and consequently of maize grain yield, via introduction of the *Hap2* allele or enhancement of *KNR6* expression using ear-specific promoters.

In summary, we cloned *qKNR6*, a maize EL and yield QTL, and found that the causal locus encodes a serine/threonine protein kinase. Overexpression of *KNR6* and introgression of alleles lacking the polymorphic *Harbinger*-like TE and an LTR retrotransposon in *KNR6* significantly enhanced grain yield. We confirmed the kinase activity of KNR6, and provided in vitro evidence that it could phosphorylate an interacting Arf GTPase-activating protein (AGAP) to regulate ear development. These results suggest the potential application of *KNR6* and *AGAP* to enhance the grain yield of maize hybrids.

## Methods

### Fine mapping of *qKNR6*

Over 28,000 F_2_ individuals derived from the NIL^*qKNR6*^ × NIL^*qknr6*^ were genotyped with ten markers (Supplementary Data 3) within the *qKNR6* interval to identify the recombinants. The heterozygous recombinants were selfed to develop into families, and each family planted more than 200 individuals for progeny testing to detect the allele effect. Meanwhile, the homozygous recombinants were selectively selfed to develop homozygous lines (RL-1 to RL-10) by marker-assisted selection. A total of ten recombinant lines were planted at Wuhan (30°N, 114°E), China, in 2015 and 2016 spring using a randomized block design with three replicates. Each plot consisted of 11 individuals grown in a single row with 3 m in length, spacing of 0.3 m between plants, and 0.6 m between rows. Seven to nine competitive individuals were harvested in a plot, and subsequently air-dried to measure the EL (cm), KNR, KRN, ear diameter (ED, cm), and EW (g). The difference significance was examined using the Duncan’s test.

### Amplification of candidate genes in NIL^*qknr6*^ and NIL^*qKNR6*^

Genomic DNA of NIL^*qknr6*^ and NIL^*qKNR6*^ was extracted from immature leaf tissue using the CTAB method, the full-length DNA sequence of *Zm00001d036601* and *Zm00001d036602* was separately amplified and sequenced using eight primer pairs (M9– M16) in NIL^*qknr6*^, and four primer pairs (M15–M18) in NIL^*qKNR6*^, three primer pairs (G678-1 to G678-3) were used to amplify *Zm00001d036601* (Supplementary Data 3), the PCR products were then cloned into the pGEM-T Easy Vector (Promega, Madison, WI, USA), and three clones for each fragment were sent for sequencing (Sangon Biotech Co., Ltd., China).

### RNA extraction and quantitative reverse transcription PCR

Approximately, 0.1 g of the immature ear tissue (1.5–2 mm) was collected from each of the recombinant lines, parent lines and 105 inbred lines. Tissue samples of seedling roots, seedling leaves, internodes, mature leaves, 1-mm ears, 4-mm ears, and 2- and 4-cm tassels from B73, and SPMs, SMs, and floral meristems (FMs) from NIL^*qknr6*^ and NIL^*qKNR6*^ were collected for the extraction of total RNAs using the Pure Link Plant RNA Reagent (Ambion, Austin, TX, USA). Approximately, 1.0 μg of RNAs were reversely transcribed by the M-MLV reverse transcriptase (Life Technologies, Invitrogen, Carlsbad, CA, USA) according to the manufacturer’s instructions. Quantitative real-time PCR (qRT-PCR) was performed using the SYBR Green qRT-PCR Kit (Bio-Rad, Hercules, CA, USA) according to the manufacturer’s instructions with three biological replicates, each with three technical replicates. The maize *ACTIN* (*Zm00001d010159*) was used as the internal control. All reactions were carried out on the CFX96 Real-time system (Bio-Rad, Hercules, CA, USA). The relative expression of the gene was calculated by the 2−ΔΔCt method.

### Genetic transformation

To construct the CaMV35S-drived *KNR6* (*KNR6*-OE), the full-length CDS of *KNR6* was amplified from the pGEM-KNR6 plasmid using primers OGF05F and OGF05R (Supplementary Data 3), then digested with *Pst* I and *Nco* I. The digested product was ligated to the binary vector pCAMBIA3301. A 520_bp coding sequence (from 567_bp to 1086_bp after ATG) at the 3′ end of *KNR6* cDNA was amplified as the target of RNAi. The target sequence was amplified; PCR products were digested with two sets of restriction enzyme combinations: *Pst* I and *Spe* I for the forward sequence and *Nde* I and *Spe* I for the reverse sequence. Two reversely ligated *KNR6*-RNAi targets were separated by a 700_bp GFP sequence. The *KNR6*-RNAi construct was then ligated with the *Ubi* promoter; the *Ubi*-driven construct was cloned into the binary vector PTF102 (Supplementary Data 3). Then the vectors were transferred to *Agrobacterium* strain EHA105 and then transformed into maize-inbred line A188.

To create mutants of Arf GTPase-activating protein gene (*AGAP*), we designed guide RNAs (gRNAs) that specifically targeted GACGGATTTGAGGCCCAACA and GTGGCTCTCCAGATCCAAAA sites of *AGAP* for gRNA construct (Supplementary, Fig. 10). For the construction of the gRNA cassette, the gRNAs were cloned into vectors pENTR-gRNA1 and pENTR-gRNA2 (kindly provided by Dr. Bing Yang at Iowa State University) according to the description of Char et al.^45^. The gRNA cassettes were then mobilized to pGW-Cas9 (kindly provided by Dr. Bing Yang at Iowa State University) through Gateway recombination. The resulting Cas9/gRNA construct was transformed into *A. tumefaciens* strain EHA101, and then was introduced into immature embryos of maize-inbred line ZZC01 through *Agrobacterium*-mediated transformation^46^. All of transgenic individuals and family lines were planted at Wuhan (30°N, 114°E) in isolation conditions.

### Rapid amplification of cDNA ends

Total RNAs isolated from immature ear tissues of NIL^*qknr6*^ and NIL^*qKNR6*^ were reversely transcribed using the Fristchoice RLM-RACE kit (Ambion, Austin, TX) according to the manufacturer's instructions. The *KNR6*-specific primers designed to amplify 5ʹ- and 3ʹ-RACE-ready cDNAs are listed in Supplementary Data 3. The gel-purified second PCR products were cloned into the pGEM-T Easy Vector (Promega, Madison, WI, USA) for sequencing. Sequences from 5ʹ- and 3ʹ-RACE products were assembled to obtain full-length cDNA sequence of *KNR6*.

### Phylogenetic analysis

The amino acid sequences of the *KNR6* and its paralogs were retrieved from Gramene (http://www.gramene.org/), which were then aligned by MEGA7.0.26 for maximum likelihood method-based phylogenetic analysis^47^.

### LUC activity assay

To test the effect of TE-PAV on gene expression, a dual-LUC transient expression assay was performed in maize protoplasts^24^. A 5570_bp of the Harbinger-like TE-contained segment and a 567_bp of the intron segment in the 5′-UTR were amplified from maize-inbred lines NIL^*qknr6*^ and NIL^*qKNR6*^, respectively. The primers for amplifying these segments are listed in Supplementary Data 3. These three segments were then cloned into upstream of the mpCaMV of pGreen II 0800-LUC vector to generate the reporter constructs, respectively. Mesophyll protoplasts were isolated from the leaves of 10-day-old etiolated B73 seedlings. Subsequently, the prepared plasmids were transformed into the prepared mesophyll protoplasts using polyethylene glycol-mediated transformation^48^. Both firefly Luc and Renilla luciferase (REN) activities were measured using the Dual-Luciferase Reporter Assay System (Promega, Madison, WI, USA) according to the manufacturer’s instructions. LUC activity of each construct was measured in three biological replicates, each with two technical replicates. Relative LUC activity was calculated by normalizing the firefly LUC activity to the *Renilla* LUC activity.

### Bisulfite sequencing and Chop-PCR

The genomic DNA was separately extracted from the 2–5-mm ears of both parent lines using DNeasy Plant Mini Kit (Qiagen, Valencia, CA, USA). DNA sample was fragmented by sonication to 200–300_bp. After 3′-A addition and adapter ligation, the DNA fragments were subjected to sodium bisulfite conversion using the ZYMO EZ DNA Methylation-Gold kit (ZYMO Research, Orange County, CA, USA) according to the manufacturer's instructions. The sequencing was performed by Wuhan Genoseq Technology with Illumina Hiseq2500 (Illumina Inc., SanDiego, CA, USA). Each library was sequenced ~554 million raw reads. Clean and high- quality reads were then generated by filtering out the adapters and low-quality reads using Trimmomatic-0.33^49^. The clean reads were aligned to the maize B73 reference genome (www.maizeGDB.org) using Bismark^50^. Only perfect matches were filtered in for methylation analysis. To calculate the methylation density of cytosine, the total number of nucleotides cytosine and thymidine that overlap with each genomic cytosine site across the whole genome was calculated. The methylation level for each cytosine site was calculated by the sequencing depth divided by the number of unconverted cytosine^51^. To screen genomic regions, a sliding-window approach was used with a 200_bp window size and a 100_bp step size. For each window, the methylation level of each context (CG, CHG, or CHH) was calculated using the number of the methylated context to the total number of the respective context. A Student’s *t* test was used to estimate the significance of difference between both lines at the methylation level in the flanking regions of *KNR6*.

For Chop-PCR, 100 ng of DNA was digested for 120 min with 1 U of *Hpa* 1, *Msp* 1, and *Alu* 1 (New England Biolabs, Ipswich, MA, USA) at 37 °C, respectively, then inactivated at 80 °C for 20 min. The reaction procedure was then subjected to 30 cycles of PCR amplification using specific primers (Supplementary Data 3).

### Candidate-gene association mapping

Candidate-gene association mapping was carried out to identify the variants of *KNR6*, which were associated with KNR in a set of 224 diverse maize lines. The Harbinger-like TE PAV was assayed using the primer combinations (GP1, TEGP1, and TEGP2), and LTR-PAV was assayed using primer combinations (LTR-F1, LTR-F2, and LTR-R) (Supplementary Data 3). The *KNR6* genomic sequence was amplified using three primer pairs (GP1–GP3) in the *Hap2* lines, and four primer pairs (TEGP1, TEGP2, GP2, and GP4) in the *Hap1* lines (Supplementary Data 3). Those amplified products were then sequenced. The sequences were aligned using MAFFT version 7^52^, and were manually adjusted using BioEdit^53^. In addition, genetic variations within 100_kb region centered on *KNR6* were also retrieved from the whole-genome resequencing data of the association panel. Polymorphic sites, including SNPs, In/Dels, and PAVs, with the minor allele frequency ≥ 0.05, were extracted in TASSEL 2.1.0^54^. All phenotypes used in the study were measured in short-day (Sanya, 18.34°N, 109.62°E) in 2015 and 2016 with one replicate each, and long-day (Ezhou, 30.04°N, 114.88°E) in 2017 with three replicates. The best linear-unbiased prediction values were estimated for association analysis. The LD among polymorphic sites was calculated and then plotted by the R package LD heatmap^55^. Association analysis was performed using a mixed model^56^, considering population structure and relative kinship^57^, in TASSEL 3.0.67^54^.

### Evaluation allelic effect in different genetic backgrounds

A total of 21 lines, including 11 *Hap1* lines and 10 *Hap2* lines, were picked out from the association panel by genotyping to cross to both NIL^*qknr6*^ and NIL^*qKNR6*^, respectively. The 42 hybrids were grown at Wuhan (30.60°N, 114.30°E) in 2015 spring, using a randomized block design with three replicates. Each plot consisted of 17 individuals grown in a single row with 5 m in length and 0.6 m in width. Twelve to fifteen competitive individuals were harvested in each plot, and subsequently air-dried to measure EL (cm), KNR, and EW (g).

### Marker-assisted backcrossing of maize-inbred lines

We transferred the *Hap2* allele into two *Hap1* allele-harbored lines (Zheng58 and Chang7-2) that are widely used in Chinese maize-breeding programs, by marker-assisted backcrossing using the NIL^*qKNR6*^ as the donor. Four backcrossing generations followed by two self-crossing generations were performed. Those improved lines were phenotypically evaluated at Wuhan (30.60°N and 114.30°E) in 2017 spring. The field experiments were performed in a randomized block design with three replicates. Each plot consisted of 2 rows, and each row grows 12 individuals. The original hybrid Zheng58/Chang7-2 and improved hybrid Zheng58^*qKNR6*^/Chang7-2^*qKNR6*^ were grown at Wuhan (30.60°N and 114.30°E) in 2017 spring and Zhengzhou (34.75°N and 113.62°E) in 2017 summer. The grain yield-related traits, including EL (cm), KNR, kernel weight (KW, g), and EW (g), were measured. Post hoc test of LSD was used for the data analysis with significant threshold *p* value < 0.05.

### Protein preparation

The full-length cDNA sequence (CDS) of *AGAP* (Zm00001d038063), *KNR6*, and its several mutants *K*_*74*_*R*, *L*_*70*_*V*, *D*_*172*_*A,* and *S*_*176*_*A* were amplified by overlap PCR, and were cloned into the His-tagged recombinant protein expression vectors pET-21b, pET-28a-SUMO vector (Novagen, Madison, WI, USA), respectively. The full-length CDS of *AGAP*, *14-3-3a* (*Zm00001d003401*), and *14-3-3b* (*Zm00001d053090*) were cloned in the GST-tagged recombinant protein expression vector pGEX-4T-1. The plasmids were transformed into *E. coli* BL21 (DE3) strain. The transformed bacterial preculture was inoculated with 1 L of lysogeny broth medium supplemented with 100 µg mL^−1^ ampicillin or kanamycin at 37 °C, until the optical density measured at 600 nm reached 1 in a UV–vis spectrometer. The recombinant protein was then induced with 0.2 mM isopropyl-β-ɗ-thiogalactoside for 16 h at 16 °C. For the His-tagged recombinant proteins, the bacterial pellet was collected and homogenized in buffer A (25 mM Tris-HCl, pH 8.0, 150 mM NaCl). After sonication and centrifugation at 23,000*g* at 4 °C, the supernatant was loaded onto a column equipped with Ni^2+^ affinity resin (GE Healthcare, 17-5318-01), washed with buffer B (25 mM Tris-HCl, pH 8.0, 150 mM NaCl, and 15 mM imidazole), and eluted with buffer C (25 mM Tris-HCl, pH 8.0, 250 mM imidazole) and followed by ion exchange (Source 15Q, GE Healthcare). For the GST-tagged recombinant proteins, the supernantant was loaded onto a column equipped with Glutathione affinity resin (GE Healthcare), washed with buffer B without 15 mM imidazole, and eluted with buffer D (50 mM Tris-HCl, pH 8.0, 10 mM Glutathinone reduced). Each protein was then subjected to gel filtration chromatography (Superdex-200 10/300, GE Healthcare). The buffer for gel filtration contained 50 mM HEPES, pH 7.4, 50 mM NaCl, and 1 mM 1,4-dithiothreitol DTT. The purified proteins were stored at −20 °C in small aliquots for further experiments.

### In vitro kinase activity analysis

Approximately, 1 µg of kinase protein was incubated with 2 µg of the kinase substrates MBP (myelin basic protein, M2295, Sigma) or AGAP or 14-3-3 proteins in a 25-µL reaction system (50 mM HEPES, pH 7.4), containing 50 mM NaCl, 1 mM DTT, 10 mM MgCl_2_, 10 µM ATP, and 1 µL (10 µCi) [γ-32p] ATP at 25 °C for 1 h. After incubation, the reaction was terminated with 25 µL of 2× sodium dodecyl sulfate (SDS) loading buffer. Fusion proteins were separated on a 15% SDS-polyacrylamide gel electrophoresis (PAGE) Gel at 50 V for 30 min and 200 V for 1 h; then the gel was stained in coomassie brilliant blue overnight with decolorization. After electrophoresis, the phosphorylation status of fusion proteins was analyzed by autoradiography using a FUJI Film FLA5000 PhosphorImager (Fujifilm, Tokyo, Japan).

### Polyclonal antibody preparation and western blotting

The mouse anti-KNR6 Polyclonal antibody (Ab-KNR6) was prepared in the Gene Create Biological Engineering Co., Ltd. (Wuhan, China) using the custom peptide MSAVVAMLRGEADVDT according to standard protocols. The Cys-cross-linking antigen was used to immunize female mice four times at 1- or 2-week intervals. Approximately, 50 μg of antigens and an equal volume of Freund’s complete adjuvant (Sigma-Aldrich, St. Louis, MO, USA) were mixed and injected subcutaneously for the primary immunization, and followed by three injections with 50 μg of the same immunogen in Freund’s incomplete adjuvant at 2-week intervals. After the final immunization, polyclonal antibodies were purified from collected sera. Titration of a specific polyclonal antibody was then performed using ELISA.

Crude protein was extracted from 0.5 g of collected tissue by 0.5 mL of protein buffer (50 mM Tris-HCl, pH 7.2, 150 mM NaCl, protease inhibitor, and 1% Triton X-100), and then was mixed with 5× SDS loading buffer. The protein sample was subjected to electrophoresis on a 12% (w/v) polyacrylamide gel and transferred to a polyvinylidene fluoride membrane (Bio-Rad, Hercules, CA, USA). The membrane was incubated for 1 h at room temperature with 5% nonfat milk in phosphate-buffered saline (PBS) containing 0.05% Tween 20 (PBS-T). All antibody incubations were performed in PBS-T containing 3% nonfat milk. KNR6 was detected with Ab-KNR6 serum at a dilution of 1:2000 (v/v) overnight at 4 °C. Anti-Actin (dilution of 1:2000 (v/v)) was used as an endogenous control (A0480, Sigma). The membrane was incubated for 1 h with a goat anti-mouse immunoglobulin horseradish peroxidase-conjugated secondary antibody (12-349, Sigma) at a dilution of 1:3000 (v/v) to visualize the signal.

### Pull-down assay

For the His-resin pull-down assay, ~50 µg of the purified proteins were mixed and incubated for 3 h at 4 °C, and then subjected to pull-down assay with Ni^2+^ affinity resin (GE Healthcare) for 1 h at 4 °C. The beads were collected by centrifugation and then washed five times with buffer containing 25 mM Tris-HCl, pH 8.0, 150 mM NaCl, and 0.1% Triton X-100. Subsequently, the proteins bound on the beads were eluted with buffer C (25 mM Tris-HCl, pH 8.0, 250 mM imidazole). The elution was added 5× SDS sample buffer and boiled at 95 °C for 10 min, and then subjected to SDS-PAGE and immunoblot with anti-GST (Abcam, ab187949) and anti-His (Abcam, ab184607) antibodies.

### Immunoprecipitation–mass spectrometry

Approximately, 5-mm ears of B73 were collected and grinded in a mortar using liquid nitrogen. The frozen powder was mixed with ice-cold extraction buffer (50 mM Tris-HCl, 150 mM NaCl with protease inhibitors, 1 mM phenylmethanesulfonyl fluoride, and 1% Triton ×100). The total proteins were placed on ice for 30 min and centrifuged at 10,000*g* for 10 min at 4 °C. Approximately, 1 mM disuccinimidyl suberate (DSS, MAN0011240, Thermo Fisher, Waltham, MA, USA) was used for Ab-KNR6 antibody and A/G magnetic beads (MAN0015742, Thermo Fisher, Waltham, MA, USA) cross-linking according to the manufacturer’s instructions. The total proteins were incubated with the beads overnight at 4 °C. The beads were magnetically separated and washed twice, and were then heated in 100 µL of 1× SDS-sample buffer at 95 °C. The immunocomplexes were analyzed by mass spectrometry in the Omics Space (Shanghai, China) (www.omicsspace.com). The mass spectrometry data was searched against the protein database of the B73 AGPv4 pep.fastas using Proteome Discoverer software, version 2.1 (Thermo Fisher, Waltham, MA, USA). The raw data was searched using SEQUEST HT algorithm in Proteome Discoverer version 2.1 and Mascot search nodes in Mascot 2.3. Peptide precursor mass tolerance was set at 20 ppm, and MS/MS tolerance was set at 0.1 Da. Searches were initiated with trypsin as the enzyme, allowing a maximum of two missed cleavages with a minimum peptide length of six amino acids. Other parameters include the static carbamidomethylation of cysteine and variable modifications of oxidation on methionine as variable modification. High-confident peptides were identified at a false-discovery rate of 1% using Proteome Discoverer 2.1.

### Firefly LUC complementation imaging

The full-length CDS of *KNR6* and *AGAP* was constructed into the 35 S::NLuc and 35 S::CLuc by recombinant enzyme *Sal* I, respectively. The constructs were introduced into *Agrobacterium tumefaciens* strain GV3101. Bacterial suspensions harboring the indicated constructs were infiltrated into fully expanded leaves of *Nicotiana benthamiana* plants using a needleless syringe^58^. GV3101 strains harboring KNR6-NLuc and AGAP-CLuc were co-infiltrated in *N. benthamiana* leaves. GV3101 strains harboring NLuc instead of KNR6-NLuc and CLuc instead of AGAP-CLuc were co-infiltrated as a control. After infiltration, plants were immediately covered with plastic bags and placed at 23 °C for 48 h before bag removal. Plants were then incubated at 28 °C with 16-/8-h light/dark cycles before the Luc activity was measured. Two days after inoculation, 1 mM luciferin was sprayed onto the inoculated leaves. The sprayed leaves were then kept under dark for 6 min to quench the fluorescence. A low-light cooled CCD imaging apparatus (Carestream Health, Rochester, NY, USA) was used to capture the Luc image.

### In situ hybridization

Immature B73 ears were collected and fixed in a solution containing 5% formalin, 50% ethanol, and 5% acetic acid (FAA) for 16 h at 4 °C, and then replaced with 70% ethanol twice and dehydrated with an ethanol series, substituted with xylene, embedded in Paraplast Plus (Sigma, St. louis, MO, USA), and sectioned to 7–8 μm. For making sense and antisense RNA probes, gene-specific primer sets were used to amplify *KNR6* and *AGAP* (Supplementary Data 3). The amplified products were cloned into pSPT18 (Roche, Basel, Switzerland) and linearized with *Hin*d III and *Eco*R I, respectively. The sense and antisense probes were separately synthesized by RNA polymerase using SP6 or T7 primer with digoxigenin-UTP as the label. Hybridization was performed according to Jackson (1991)^59^ with the addition of 8% polyvinyl alchol to the detection buffer. Slides were exposed for 12–15 h before mounting and imaging, and were visualized under a microscope (Nikon eclipse 80i, Japan).

### Reporting summary

Further information on research design is available in the Nature Research Reporting Summary linked to this article.

## Supplementary information

Supplementary Information

Peer Review File

Reporting Summary

Description of Additional Supplementary Files

Supplementary Data 1

Supplementary Data 2

Supplementary Data 3

## Data Availability

A reporting summary for this article is available as a Supplementary Information file. Data supporting the findings of this work are available within the paper and its Supplementary Information files. The datasets generated and analyzed during the current study are available from the corresponding author upon request. Sequence data in this study can be found at NCBI under nucleotide accessions MG582650, MG664870–MG665220, and Sequence Read Archive project number PRJNA587806. The source data underlying Figs. 1b–d, 2c, d, g, h, 3e–h, 4c–f, i, j, and 5d–g, as well as Supplementary Figs. 1f, 10c, d, 11, 12c, d are provided as a Source Data file.

## Source Data

Source Data
