# Supplementary material for: A serine/threonine protein kinase encoding gene KERNEL NUMBER PER ROW6 regulates maize grain yield
Source: Nat Commun. 2020 Feb 20;11:988. doi: 10.1038/s41467-020-14746-7 (PMC7033126; doi:10.1038/s41467-020-14746-7)
Supplement: Supplementary file 1 — Supplementary Information [file 41467_2020_14746_MOESM1_ESM.pdf]

A serine/threonine protein kinase encoding gene *KERNEL NUMBER PER ROW6* regulates maize grain yield

Jia *et al.*

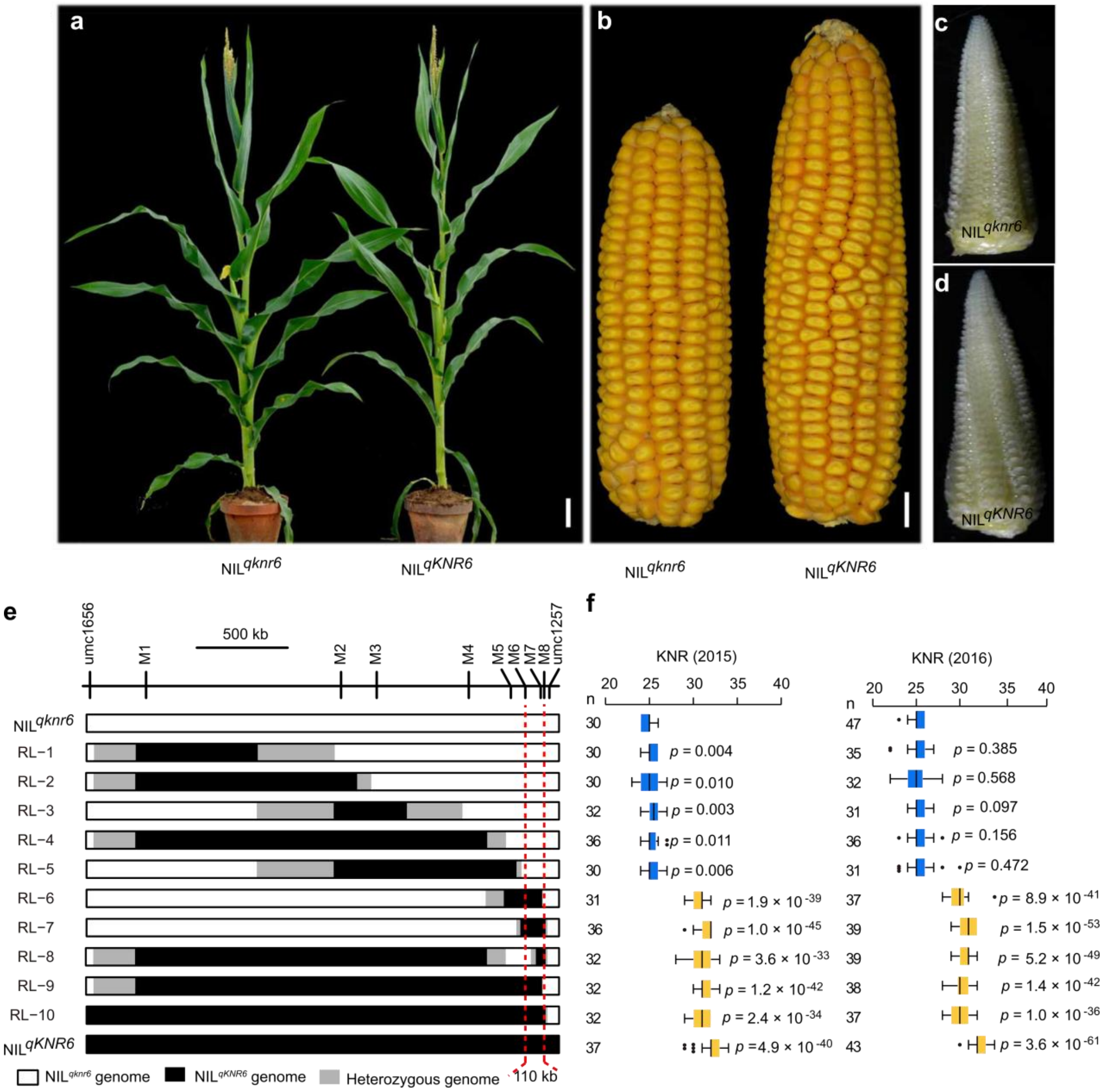

Supplementary Figure 1. Plant and ear status of two parental lines and kernel number per row of the recombinant lines. a-d Plant status (**a**) and ears (**b**) and developing ear inflorescences (**c** and **d**) of two parental lines. **e** Genotypes of 10 recombinant lines. **f** Box-and-whisker plots of kernel number per row of 10 recombinant lines and two parental lines evaluated at Wuhan in 2015 and 2016. Each box represents the median and interquartile range. Whiskers extend to maximum and minimum values. Data are shown as the mean ± s.d. and the difference significance is examined by the one-way ANOVA. n, the number of eras examined. Scale bar = 10 cm in (**a**), and 1 cm in (**b**). Source data underlying Supplementary Figure 1f are provided in a Source Data file.

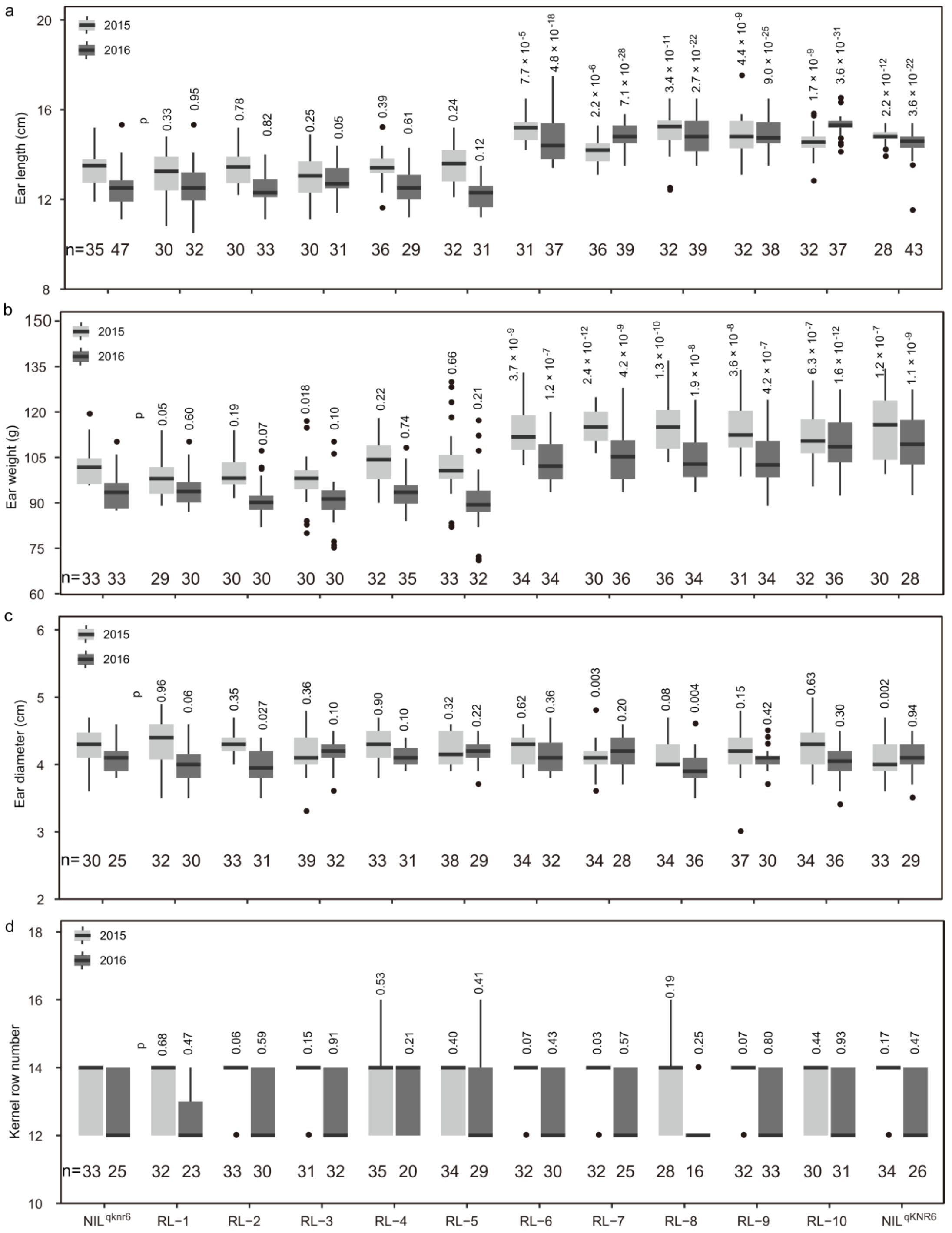

Supplementary Figure 2. Phenotypes of ear-related traits of the recombinant lines. a-d Box-and-whisker plots of ear length (EL, cm) (***a***), ear weight (EW, g) (***b***), ear diameter (ED, cm) (***c***), kernel row number (KRN) (***d***) of recombinant lines and two parents (NIL^{qknr6} and NIL^{qKNR6}). Each box represents the median and interquartile range. Whiskers extend to maximum and minimum values. Phenotypes of lines are evaluated using a randomized block design with three replicates at Wuhan in 2015 spring and 2016 spring, respectively. The difference significance is examined by the one-way ANOVA. n, the number of eras examined.

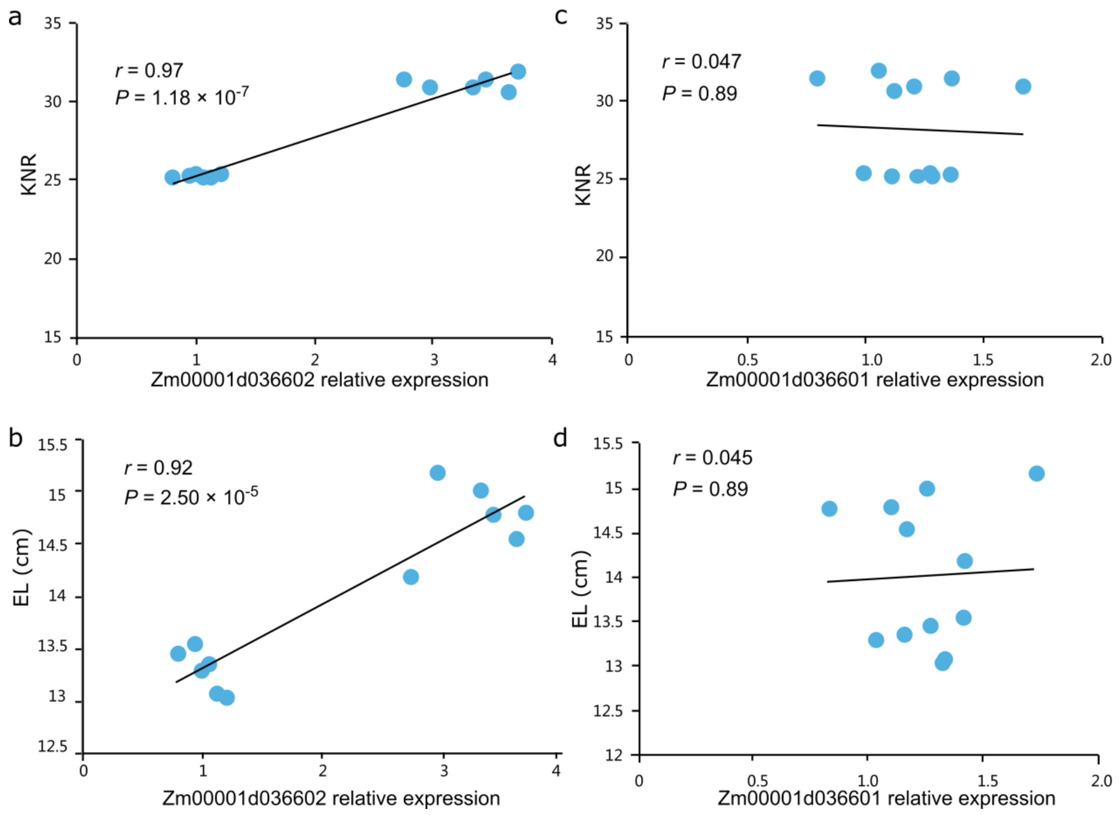

Supplementary Figure 3. Correlation between the expression levels of two candidate genes with KNR and EL in 10 recombinant lines. **a** and **b** Pearson correlation between *Zm00001d036602* expression levels with kernel number per row (KNR) (**a**) and ear length (EL) (**b**). **c** and **d** Pearson correlation between *Zm00001d036601* expression levels with kernel number per row (**c**) and kernel number per row (**d**). *P*-values are determined by the two-tailed Student's t-test to establish if the correlation coefficient is significantly different from zero. n = 12.

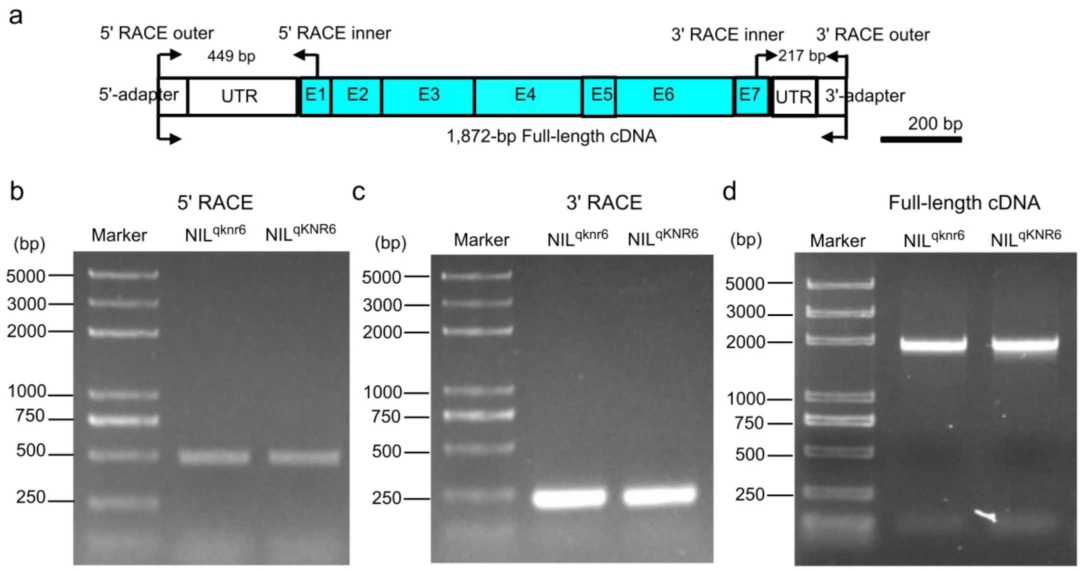

Supplementary Figure 4. Rapid amplification of *KNR6* cDNA ends in NIL^{qknr6} and NIL^{qKNR6}. a A graphical representation of positions of primers for Rapid amplification of *KNR6* cDNA ends. E: Exon. UTR: untranslated region. **b** The gel electrophoresis results of 5′-RACE of *KNR6* in the NIL^{qknr6} and NIL^{qKNR6}. **c** The gel electrophoresis results of 3'-RACE of *KNR6* in the NIL^{qknr6} and NIL^{qKNR6}. **d** The gel electrophoresis results of full-length cDNA amplification of *KNR6* in the NIL^{qknr6} and NIL^{qKNR6}, respectively.

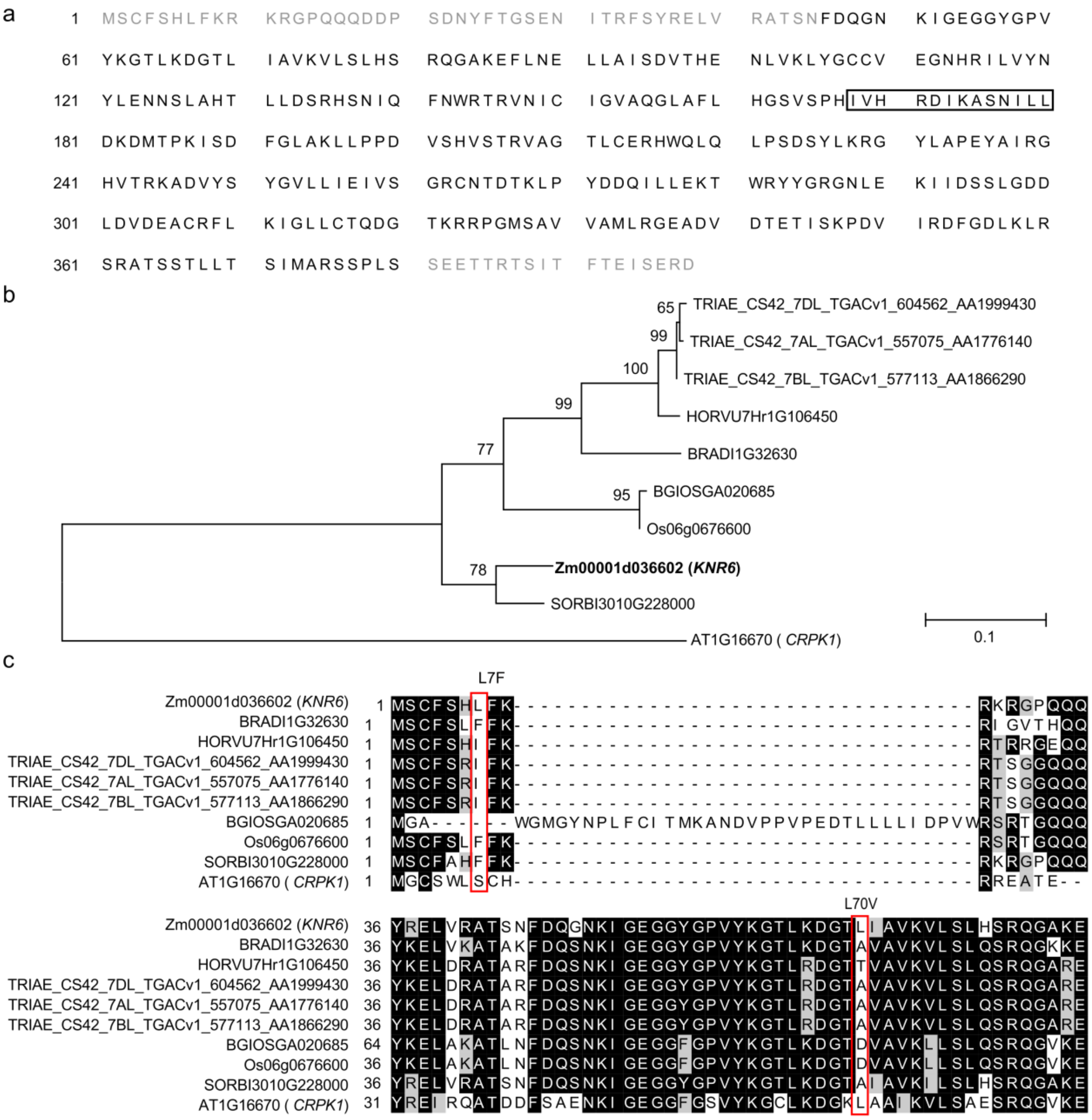

Supplementary Figure 5. Characteristics of KNR6 protein. a Putative amino acid sequence of KNR6. The black amino acids show the kinase domain from 46th to 380th amino acids, and the black box points out the active site from 168th to 180th amino acid. **b** Phylogenetic tree of KNR6 across grass species. Protein sequences are downloaded from the Gramene (http://www.gramene.org/). **c** The 7th and 70th amino acids are not conserved across grass species.

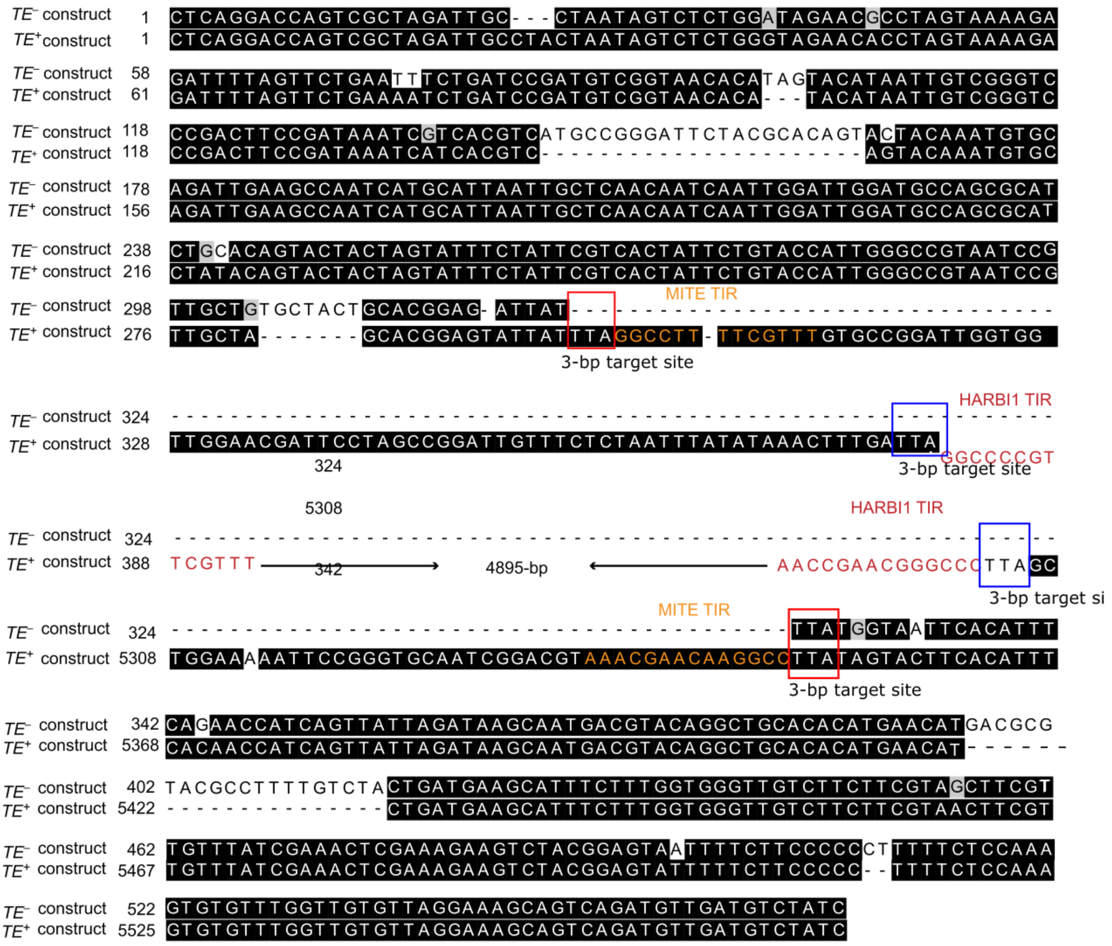

Supplementary Figure 6. Sequences of alleles used for the transient expression assay in maize leaf protoplasts. The sequence length of maize *TE*$^{-}$ and *TE*$^{+}$ constructs is 567_bp and 5570_bp, respectively. Identical nucleotides are indicated by dark boxes.

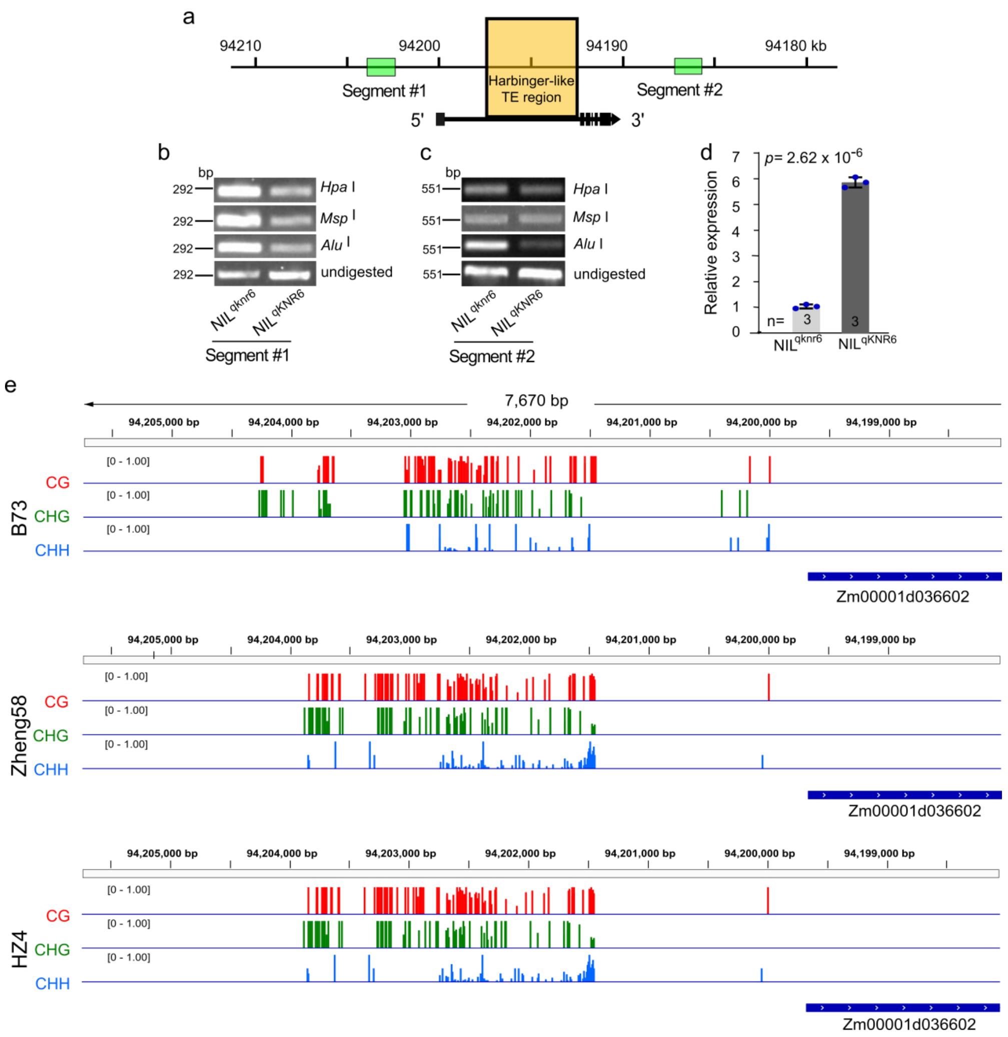

Supplementary Figure 7. DNA methylation of *KNR6* in two parent lines and three lines with the *Harbinger*-like transposable element. a A diagram on chromosome locations of *KNR6*, the Harbinger-like TE and two amplified segments. **b** and **c** Electropherogram of Chop-PCR products amplified in the segment #1 (**b**) and the segment #2 (**c**). Undigested genomic DNA and genomic DNA digested with methylation-sensitive enzyme *Hpa*1, *Msp*1 and *Alu*1 are subjected to PCR amplification using segment-specific primers, respectively. **d** Relative expression of *KNR6* in two parental lines. Expression level is measured by qPCR with three biological replicates and each with three technical replicates. Maize *Actin* gene (*Zm00001d010159*) is used as internal control. Data are shown as the mean ± s.d., and significance is estimated by the two-tailed Student's t-test. **e** DNA methylation of a 7,670_bp region on the 5'-UTR of KNR6. The methylation of three contexts (CG, CHG, and CHH) are identified in three inbred lines (B73, Zheng58, HZ4 (Huangzao 4)) that have the insertion of the harbinger-like TE in the 5'-UTR intron of *KNR6*.

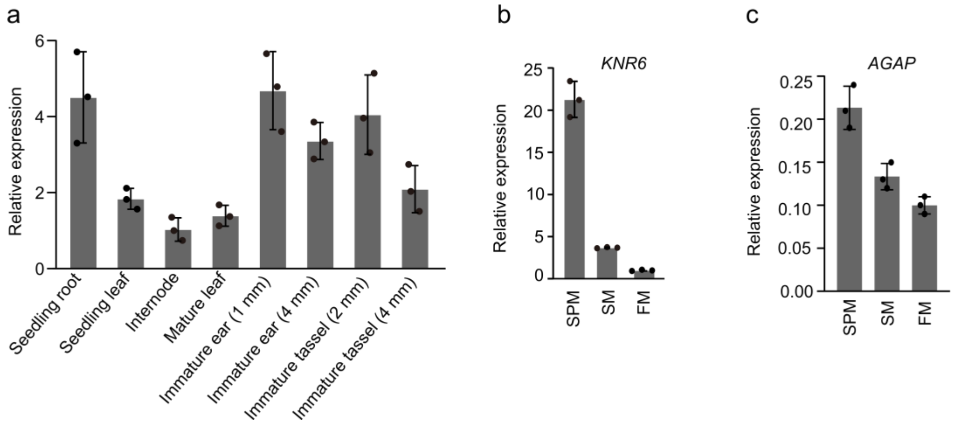

Supplementary Figure 8. Expression pattern of *KNR6 and AGAP*. a Relative expression level of *KNR6* in seedling root, seedling leaf, internode, 1 mm and 4 mm ears, and 2 mm and 4 mm tassels by qPCR, respectively. **b** and **c** Expression of *KNR6* (**b**) and *AGAP* (**c**) in spikelet-paired meristem (SPM), spikelet meristem (SM) and floret meristem (FM). Expression level is measured by qPCR with three biological replicates, each with three technical replicates. Maize *Actin* gene (*Zm00001d010159*) is used as internal control. Data are shown as the mean ± s.d., and significance is estimated by the two-tailed Student's t-test..*KNR6*: *KERNEL NUMBER PER ROW6. AGAP:* Arf-GTPase Activating Protein.

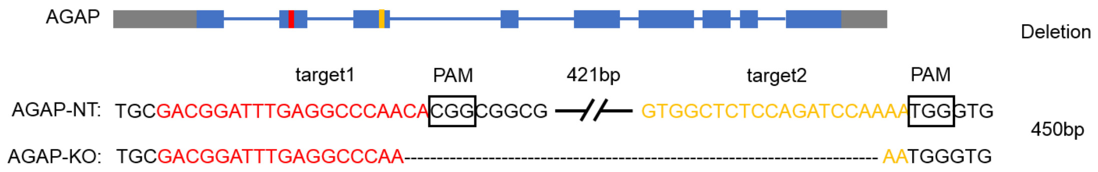

Supplementary Figure 9. The guide RNAs of AGAP and the 450_bp deletion in AGAP knockout line. The two target sites of gRNAs are marked red and orange respectively. The protospacer-adjacent motif (PAM) is marked with box, and deletions are indicated by dashes. The two gRNAs sites are located in the second and third exon of AGAP, respectively. The 450_bp deletion which leads to premature transcriptional termination of AGAP at the 60 amino acid is created between the two gRNAs.

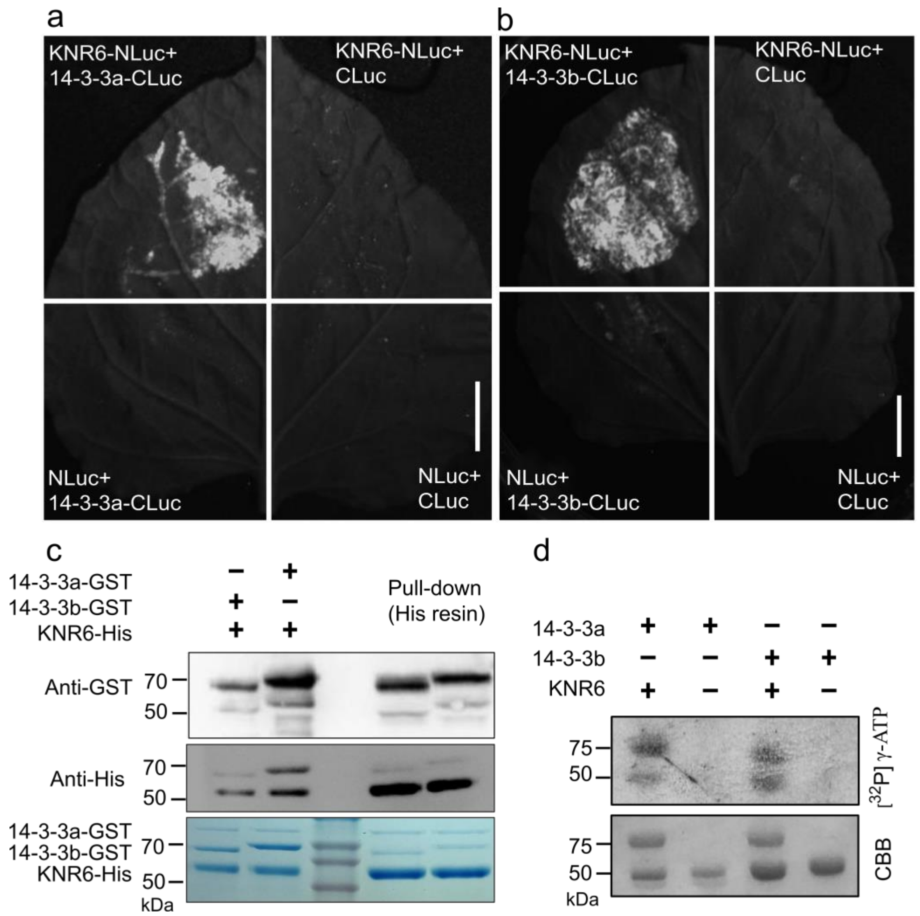

Supplementary Figure 10. The interaction and phosphorylation assay between KNR6 and 14-3-3 proteins. a and **b** Confirmation of interactions between KNR6 and 14-3-3 proteins including Zm00001d053090(**a**) and Zm00001d003401(**b**) by firely luciferase complementation imaging assay. **c** KNR6 interacts with 14-3-3 proteins in His pull-down assay.14-3-3a in (**a,c**) represents Zm00001d053090; 14-3-3b in (**b,c**) represents Zm00001d003401. Recombinant His-KNR6 protein was incubated with GST or GST-tagged 14-3-3 proteins bound to His resin at 4 °C. The eluates were resolved by SDS-PAGE and blotted using anti-GST and anti-His antibodies. **d** KNR6 phosphorylates 14-3-3 proteins. 14-3-3a shows Zm00001d053090, and 14-3-3b represents Zm00001d003401. MW, molecular weight. Scale bars = 1 cm in (**a**,**b**). A plus sign (+) means that a given substrate has been added; Minus sign (–) A minus sign indicates that a given substrate has not been added. Source data underlying Supplementary Figure 10c and 10d are provided in a Source Data file.

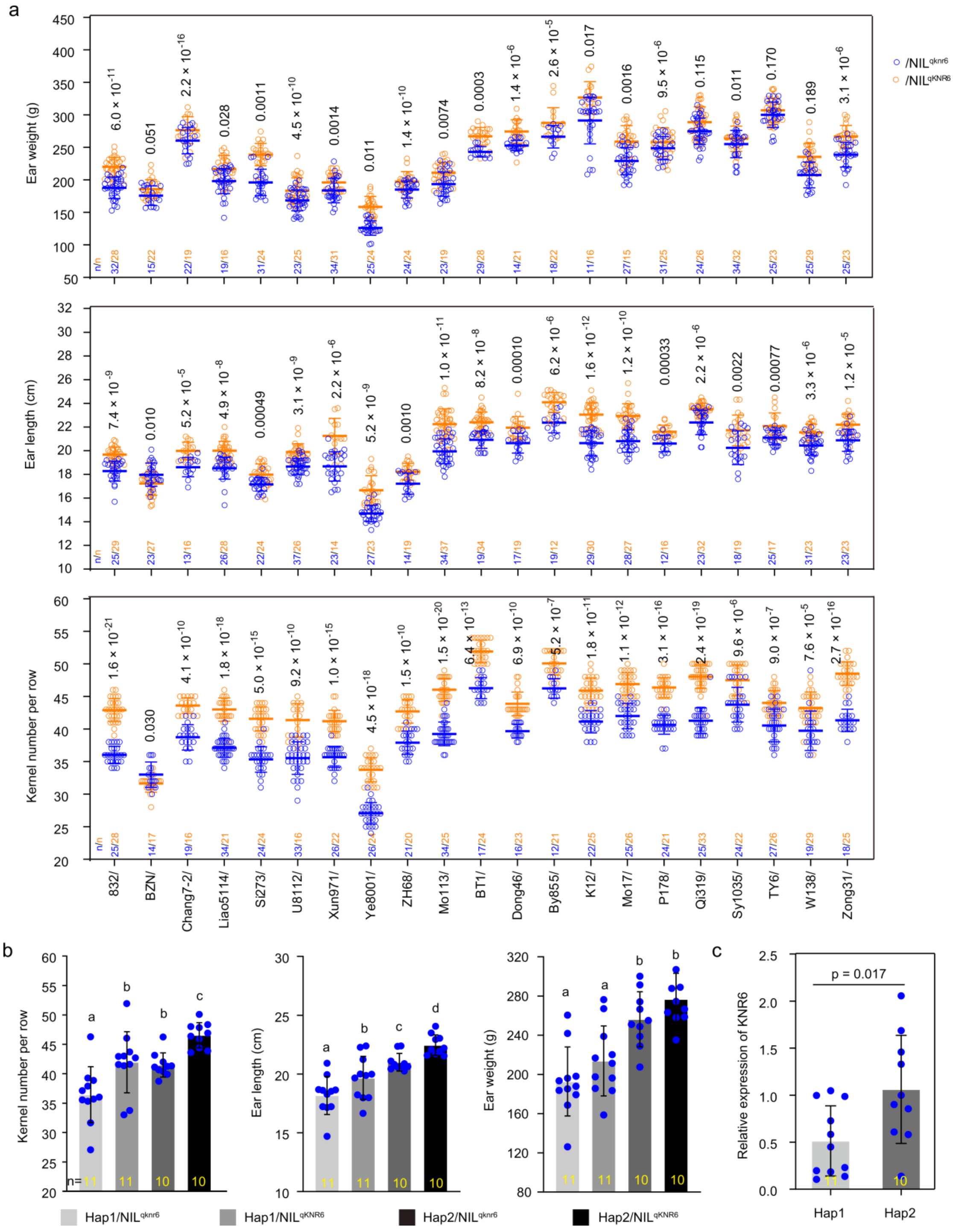

Supplementary Figure 11. Genetic effect of the transposable element insertion in *KNR6*. a Scatter plots of kernel number per row (KNR),ear length (EL) and ear weight (EW) of the hybrids developed by 21 inbred lines separately crossing to NIL^{qknr6} and NIL^{qKNR6}. Data are shown as the mean ± s.d., and dots extend to the maximum and minimum values are showed. P-value is estimated using a two-tailed Student's t-test. **b** Average performance of kernel number per row (KNR),ear length (EL) and ear weight (EW) in hybrids. The twenty-one inbred lines can be clustered

into two groups based on the haplotypes of *KNR6*: 11 *Hap1* lines and 10 *Hap2* lines. **c** Expression levels of KNR6 in 10 *Hap2* lines and 11 *Hap1* lines. Data are shown as the mean ± s.d. and Tukey HSD test is used and statistical differences ($p < 0.05$) are indicated by different letters. n, the number of ears examined. Source data are provided in a Source Data file.

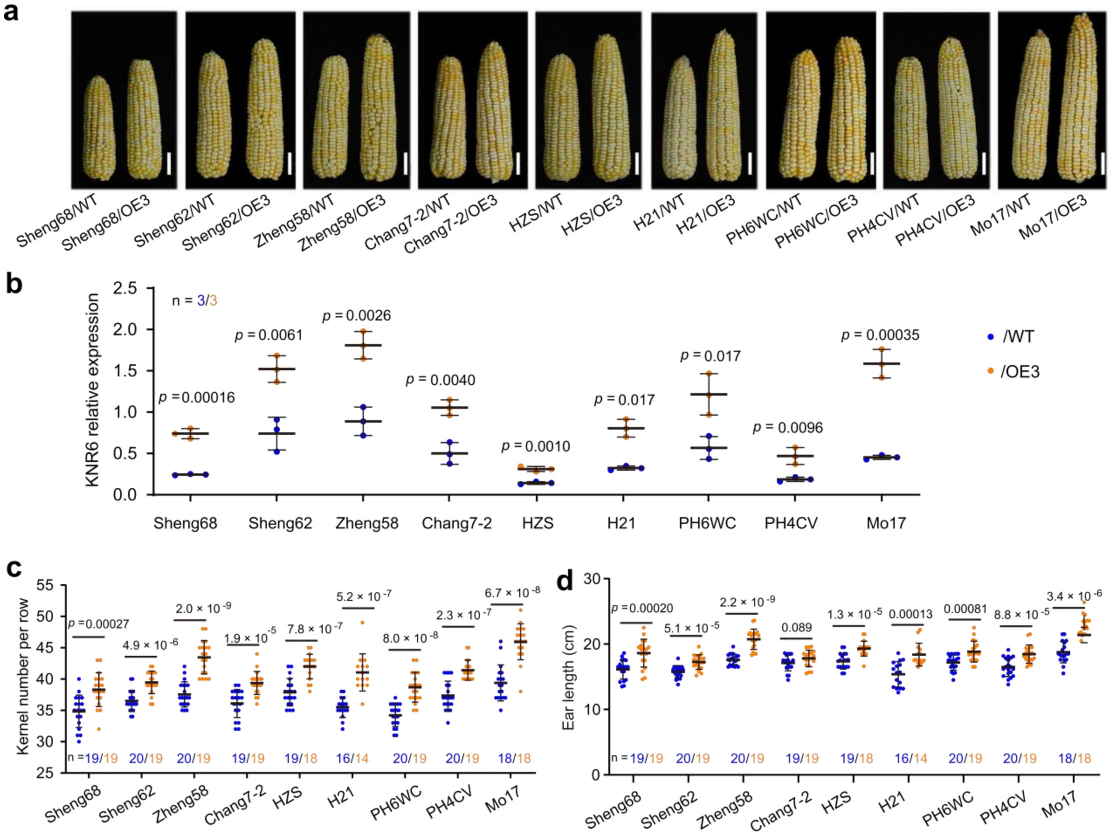

Supplementary Figure 12. Phenotypes of ear traits and flowering time in hybrids developed from KNR6-overexpressed line KNR6-OE3. a Ear images of eighteen hybrids developed from nine inbred (Sheng68, Sheng62, Zheng58, Chang7-2, HZS, H21, PH6WC, PH4CV, Mo17) crossing to *KNR6* overexpression line KNR6-OE3 and non-transgenic line (WT), respectively. Scale bar = 3 cm. **b** Expression level of *KNR6* in the immature ears of hybrids. **c** and **d** Scatter plots of kernel number per row (KNR) (**c**) and ear length (EL) (**d**) in the eighteen hybrids. Data are shown as mean± s.d., and dots extend to maximum and minimum values; p-value is estimated using a Student's T-Test. n, the number of ears examined. Source data underlying Supplementary Figure 12c and 12d are provided in a Source Data file.

Supplementary Table 1. Phenotypes of agriculturally important traits of two parent lines.

Trait	NIL^{qKNR6}	Sample size	NIL^{qkmr6}	Sample size	*p*-value
Floret number/row	40.5±1.48	30	33.1±1.07	30	6.73×10^{-30}
Tassel length (cm)	24.0±2.21	28	20.3±3.01	32	8.62×10^{-5}
Ear weight (g)	92.9±10.7	28	79.3±11.2	32	3.92×10^{-8}
Kernel weight (g)	80.3±8.45	28	65.4±9.97	32	3.91×10^{-8}
100-Kernel weight (g)	24.6±1.31	28	25.2±1.36	32	0.19
Kernel row number	12.7±1.27	28	13.1±1.09	32	0.06

The *p*-value is estimated using the two-tailed one-way ANOVA

Supplementary Table 2. Phenotypes of ear-related traits of transgenic lines.

Trait	Kernel row number	Ear diameter (cm)	Tassel length (cm)	Tassel branch number
NT	13.9±0.4	3.4±0.22	30.7±1.84	10.37±1.23
OE3	14.1±0.49	3.6±0.25	32.7±0.95	11.08±1.28
Δ	0.2	0.2	2	0.71
p-value	NS	1.51×10^{-5}	3.08×10^{-8}	0.0085
N	43/48	43/48	43/48	43/48
NT	13.8±0.60	3.7±0.22	30.7±1.13	10.2±0.43
OE4	14.0 ±0.41	3.6±0.21	32.8±0.64	11.2±0.68
Δ	0.2	-0.1	2.1	1
p-value	0.1	0.07326	3.54×10^{-8}	3.65×10^{-7}
N	21/27	21/27	21/27	21/27
NT	14.0±0.32	3.4±0.15	30.9±1.50	10.24±1.60
RNAi-1	13.8±0.7	3.4±0.31	28.3±2.60	8.24±1.15
Δ	-0.2	0	-2.6	-2
p-value	0.1	0.41	0.0009	0.0003
N	17/17	17/17	17/17	17/17
WT	13.8±0.63	3.5±0.12	30.47±1.37	9.68±0.82
RNAi-2	13.8±0.62	3.4±0.11	28.38±0.69	8.85±0.82
Δ	0	-0.1	-2.09	-0.83
p-value	0.48	0.0043	1.56×10^{-6}	0.0035
N	19/20	19/20	19/20	19/20

NT: non-transgenic plant. OE: transgenic plant with KNR6 overexpressed. RNAi: transgenic plants with KNR6 silenced via RNA interference. Δ: phenotype difference between transgenic line and respective non-transgenic line. Phenotype value is mean ± s.d. N: sample size for non-transgenic plants/transgenic plants. The *p*-value is estimated by a Student's t-test.

Supplementary Table 3. Genetic effect on allele substitution at KRN6 for important traits.

Line	N	KNR	*p*-value	EL (cm)	*p*-value	EW (g)	*p*-value
Chang7-2	57	23.8±1.3	4.1×10^{-26}	10.4±0.9	1.4×10^{-15}	76.5±12.5	1.8×10^{-4}
Chang7-2^{qKNR6}	43	27.5±1.2		11.9±0.6		86.8±10.9	
Zheng58	63	24.7±1.2	2.6×10^{-29}	13.2±1.8	2.4×10^{-13}	82.0±8.8	3.2×10^{-7}
Zheng58^{qKNR6}	89	27.7±1.5		15.3±1.2		96.1±7.27	

Phenotype are evaluated in BC_4F_2 families grown at Wuhan(30°N, 114°E) in 2017 spring,Value is presented as mean±s.d.

KNR: kernel number per row. EL: ear length. N: sample size.

$Line^{qKNR6}$ presences the line improved using the *KNR6* Haplotype2 allele.

The difference significance is examined using the two-tailed one-way ANOVA
