# Supplementary material for: A serine/threonine protein kinase encoding gene KERNEL NUMBER PER ROW6 regulates maize grain yield
Source: Nat Commun. 2020 Feb 20;11:988. doi: 10.1038/s41467-020-14746-7 (PMC7033126; doi:10.1038/s41467-020-14746-7)
Supplement: Supplementary file 3 — Reporting Summary [file 41467_2020_14746_MOESM3_ESM.pdf]

nature research

Corresponding author(s): NCOMMS-19-28382A

Last updated by author(s): Jan 18, 2020

Reporting Summary

Nature Research wishes to improve the reproducibility of the work that we publish. This form provides structure for consistency and transparency in reporting. For further information on Nature Research policies, see Authors & Referees and the Editorial Policy Checklist .

Statistics

For all statistical analyses, confirm that the following items are present in the figure legend, table legend, main text, or Methods section.

n/a	Confirmed	
☐	☒	The exact sample size (*n*) for each experimental group/condition, given as a discrete number and unit of measurement
☐	☒	A statement on whether measurements were taken from distinct samples or whether the same sample was measured repeatedly
☐	☒	The statistical test(s) used AND whether they are one- or two-sided<br>*Only common tests should be described solely by name; describe more complex techniques in the Methods section.*
☐	☒	A description of all covariates tested
☐	☒	A description of any assumptions or corrections, such as tests of normality and adjustment for multiple comparisons
☐	☒	A full description of the statistical parameters including central tendency (e.g. means) or other basic estimates (e.g. regression coefficient) AND variation (e.g. standard deviation) or associated estimates of uncertainty (e.g. confidence intervals)
☐	☒	For null hypothesis testing, the test statistic (e.g. *F*, *t*, *r*) with confidence intervals, effect sizes, degrees of freedom and *P* value noted<br>*Give P values as exact values whenever suitable.*
☒	☐	For Bayesian analysis, information on the choice of priors and Markov chain Monte Carlo settings
☒	☐	For hierarchical and complex designs, identification of the appropriate level for tests and full reporting of outcomes
☐	☒	Estimates of effect sizes (e.g. Cohen's *d*, Pearson's *r*), indicating how they were calculated

Our web collection on statistics for biologists contains articles on many of the points above.

Software and code

Policy information about availability of computer code

Data collection	1. The amino acid sequences of the KNR6 and its paralogs were retrieved from Gramene (http://www.gramene.org/).<br>2. The Bisulfite sequencing was performed by Wuhan Genoseq Technology with Illumina Hiseq2500 (Illumina Inc., SanDiego, CA, USA). Each library was sequenced approximately 554 million raw reads.<br>3. Phenotype collection of two parent lines: to count the number of florets, 30 ear inflorescences were examined in each line; to identify the ear length and kernel number per row, 32 ears from NILqknr6 and 28 ears from NILqKNR6 were phenotyped.<br>4. Phenotype collection of recombinant lines: a total of 10 recombinant lines were planted at Wuhan (30°N, 114°E) in 2015 and 2016 spring using a randomized block design with three replicates. Each plot consisted of 11 individuals grown in a single-row with 3 m in length, spacing of 0.3 m between plants and 0.6 m between rows. Thirty to thirty seven ears were phenotyped in 2015, and thirty one to forty seven ears were phenotyped in 2016.<br>5. Phenotype collection of transgenic lines: All of transgenic individuals and family lines were planted at Wuhan (30°N, 114°E) in isolation conditions. Variable numbers of individuals were used for phenotyping the ear length and kernel number per row, 36 individuals for each of NT1 and RNAi-1, 34 for NT2, 36 for RNAi-2, 34 for NT3, 31 for OE3, 32 for NT4, 34 for OE4.<br>6. Phenotype collection of association population: phenotype of association panel were measured in short-day (Sanya, 18.34°N, 109.62°E) in 2015 and 2016 with one replicate each, and long-day (Ezhou, 30.04°N,114.88°E) in 2017 with three replicates. The best linear unbiased prediction values were estimated for association analysis.
Data analysis	1. Phylogenetic analysis: the amino acid sequences of the KNR6 and its paralogs were aligned by MEGA7.0.26 using maximum likelihood methods.<br>2. Association mapping: the DNA sequences were aligned using MAFFT version 7, and were manually adjusted using BioEdit. Polymorphic sites, including SNPs and In/Del's, with the minor allele frequency ≥0.05 were extracted in TASSEL 2.1.0. Association analysis was performed using a mixed model, considering population structure and relative kinship, in TASSEL 3.0.67.<br>3. Bisulfite Sequencing data analysis: clean and high quality reads were then generated by filtering out the adapters and low-quality reads using Trimmomatic-0.33. The clean reads were aligned to the maize B73 reference genome (www.maizeGDB.org) using Bismark. Only perfect matches were filtered in for methylation analysis. To calculate the methylation density of cytosine, the total number of nucleotides cytosine and thymidine that overlap with each genomic cytosine site across the whole genome was calculated. The

methylation level for each cytosine site was calculated by the sequencing depth divided by the number of unconverted cytosine. To screen genomic regions, a sliding-window approach was used with a 200-bp window size and a 100-bp step size. For each window, the methylation level of each context (CG, CHG or CHH) was calculated using number of methylated context to total number of respective context. A Student's t-test was used to estimate the significance of difference between both lines at methylation level in the flanking regions of KNR6.
4. Gene expression analysis: Quantitative real-time PCR (qRT-PCR) was performed using the SYBR Green qRT-PCR Kit (Bio-Rad, Hercules, CA, USA) according to the manufacturer's instructions with three biological replicates, each with three technical replicates. The maize ACTIN (Zm00001d010159) was used as the internal control. Relative expression of gene was calculated by 2−ΔΔCt method.
5. Statistical analysis: difference significance of phenotype between lines was estimated by the one-way ANOVA; difference significance of gene expression was estimated by the two-tailed Student's T-test; difference significance of phenotype among hybrids was estimated by the Tukey HSD test.

For manuscripts utilizing custom algorithms or software that are central to the research but not yet described in published literature, software must be made available to editors/reviewers. We strongly encourage code deposition in a community repository (e.g. GitHub). See the Nature Research guidelines for submitting code & software for further information.

Data

Policy information about availability of data

All manuscripts must include a data availability statement. This statement should provide the following information, where applicable:

- Accession codes, unique identifiers, or web links for publicly available datasets
- A list of figures that have associated raw data
- A description of any restrictions on data availability

Data availability:
1) Sequence data in this study can be found at NCBI under nucleotide accessions: MG582650 (https://www.ncbi.nlm.nih.gov/nuccore/MG582650), MG664870–MG665220 (www.ncbi.nlm.nih.gov/ nuccore/MG664870 to MG665220), and Sequence Read Archive under project number:PRJNA587806 (https://www.ncbi.nlm.nih.gov/Traces/study/?acc= PRJNA587806).
2) The source data underlying Figs 1b-d, 2c–d, 2g-h,3e-h,4c-f,4i-j,5d-g and Supplementary Figs 1f, 10c-d, 11a-c, and 12c-d are provided as a Source Data file.

Field-specific reporting

Please select the one below that is the best fit for your research. If you are not sure, read the appropriate sections before making your selection.

☒ Life sciences ☐ Behavioural & social sciences ☐ Ecological, evolutionary & environmental sciences

For a reference copy of the document with all sections, see nature.com/documents/nr-reporting-summary-flat.pdf

Life sciences study design

All studies must disclose on these points even when the disclosure is negative.

Sample size	1. Phenotype evaluation of parent lines: 30 ear inflorescences from a line were sampled to count the number of florets; to identify the ear length and kernel number per row, 32 ears from NILqknr6 and 28 ears from NILqKNR6 were sampled.<br>2. Phenotype evaluation of transgenic lines, 36 individuals for each of NT1 and RNAi-1, 34 for NT2, 36 for RNAi-2, 34 for NT3, 31 for OE3, 32 for NT4, 34 for OE4 were sampled, respectively.<br>3. Association mapping: a total of 93 variants from 224 diverse maize inbred lines were used.<br>4. Expression association of KNR6 with traits, 105 inbred lines were used.<br>5. Phenotype evaluation of improved lines: the numbers of individuals examined from Zheng58, the improved Zheng58, Chang7-2 and the improved Chang7-2 were 57, 43, 63 and 89, respectively.
Data exclusions	For candidate-gene association mapping, those variants with minor allele frequencies < 0.05 were excluded. For methylation analysis, the adapters and low-quality reads (<30 bp) were excluded using Trimmomatic-0.33.
Replication	1. All field experiments were performed with three replicates (blocks) under at least two planting seasons.<br>2. Quantitative Real-time PCR (qRT-PCR) was performed using the SYBR Green qRT-PCR Kit (Bio-Rad, Hercules, CA, USA) with three biological replicates, and each sample with three technical replicates.<br>3. Phenotype evaluation of Hybrids was performed at two locations(Wuhan and Zhengzhou).<br>4. Firefly luciferase complementation imaging was performed with three repeated experiments.
Randomization	Field experiments: a randomized block design with three blocks were performed, and phenotypes of all traits were collected under at least two planting seasons.
Blinding	We weren't blinded to group allocation during data collection and analysis. For phenotyping the ear length and kernel number, the genotypes of individuals examined were first analyzed, and large sample size was used to eliminate random errors and ensure reliability of data.

Reporting for specific materials, systems and methods

We require information from authors about some types of materials, experimental systems and methods used in many studies. Here, indicate whether each material, system or method listed is relevant to your study. If you are not sure if a list item applies to your research, read the appropriate section before selecting a response.

Materials & experimental systems

n/a	Involved in the study
☐	☒ Antibodies
☒	☐ Eukaryotic cell lines
☒	☐ Palaeontology
☒	☐ Animals and other organisms
☒	☐ Human research participants
☒	☐ Clinical data

Methods

n/a	Involved in the study
☒	☐ ChIP-seq
☒	☐ Flow cytometry
☒	☐ MRI-based neuroimaging

Antibodies

Antibodies used	The mouse anti-KNR6 Polyclonal antibody (Ab-KNR6) was prepared in the Gene Create Biological Engineering Co., Ltd (Wuhan, China) using the custom peptide MSAVVAMLRGEADVDT according to standard protocols. The Cys-crosslinking antigen was used to immunize female mice four times at two-week intervals. Approximately 50 μg of proteins and an equal volume of Freund's complete adjuvant (Sigma, Aldrich, Germany) were mixed and injected subcutaneously for each immunization.
Validation	Titration of specific polyclonal antibody was then performed using ELISA, and western blot.
