# Supplementary material for: A serine/threonine protein kinase encoding gene KERNEL NUMBER PER ROW6 regulates maize grain yield
Source: Nat Commun. 2020 Feb 20;11:988. doi: 10.1038/s41467-020-14746-7 (PMC7033126; doi:10.1038/s41467-020-14746-7)
Supplement: Supplementary file 4 — Description of Additional Supplementary Files [file 41467_2020_14746_MOESM4_ESM.docx]

**Description of Additional Supplementary Files**

**File Name: Supplementary Data 1**

**Description:** Associations between KNR6 polymorphisms and KNR in 224 diverse maize inbred lines

**File Name: Supplementary Data 2**

**Description:** KNR6-interacting protein detected by Immunoprecipitation-Mass Spectrometry (IP-MS)

**File Name: Supplementary Data 3**

**Description:** Primers and probes used in this study
