# Supplementary material for: A serine/threonine protein kinase encoding gene KERNEL NUMBER PER ROW6 regulates maize grain yield
Source: Nat Commun. 2020 Feb 20;11:988. doi: 10.1038/s41467-020-14746-7 (PMC7033126; doi:10.1038/s41467-020-14746-7)
Supplement: Supplementary file 2 — Peer Review File [file 41467_2020_14746_MOESM2_ESM.pdf]

Reviewers' comments:

Reviewer #1 (Remarks to the Author):

Comments:

In the manuscript “KERNEL NUMBER PER ROW6 regulates grain yield by phosphorylating an Arf GTPase-activating protein in maize”, the authors established that KERNEL NUMBER PER ROW6 (KNR6) encodes a serine/threonine protein kinase that controls maize grain yield by determining pistillate floret number, ear length and kernel number per row, through fine-mapping, association mapping, expression analysis and transgenic validation. They found a Harbinger-like transposable element (TE) in the 5'-untranslated region of KNR6 is the causal variant to suppress expression of the gene and this TE has a strong impact on grain yield. Further, KNR6 regulates kernel number and grain yield by influencing AGAP phosphorylation to modulate ear inflorescence development. In this manuscript, the authors found a QTL KERNEL NUMBER PER ROW6 (KNR6) control kernel number per row (KNR) and ear length (EL), and elucidated its functional mechanism and the potential in upregulating grain yield. This finding will be of great significance to agricultural breeders. The whole manuscript is well organized and the results are presented with solid evidence and influent English. However, there are still some points need to be improved.

Major points:

1. The authors showed AGAP proteins are phosphorylated by KNR6 by phosphorylation assay in vitro, how about AGAP phosphorylation level in NILqKNR and NILqknr plants in vivo?

2. It is not suitable to draw out the role of KNR6 on 14-3-3 proteins just based on previous results from Arabidopsis and no evidence in maize. In addition, in the model of Fig. 4J, the hypothetical functions of AGAP and 14-3-3 in “auxin influx” and “physiological adaptation” is exaggerated and need to be modified.

3. The phase “meristematic activity” appeared many times in the manuscript. How to define “meristematic activity”? In Fig.1A, the yellows bars indicated the size or the shape of the inflorescence meristem. But no activity was tested using meristem markers or other methods. I think “meristematic activity” is not accurate here.

4. Fig. 5G, phenotypes of maize grown in Wuhan and Zhengzhou were compared. Grown conditions in these two positions should be described in the text.

5. “Fig. 2 Function of KNR6 on KNR and flowering time.” and “Supplementary Table 2 Phenotypes of ear-related traits and flowering time of transgenic lines.” Why mentioned “flower time”? No results of flower time were showed in these figures.

Minor points:

1. In “Supplementary Fig. 1 Plant and ear status of two parental lines and kernel number per row of the recombinant lines under two environments”, the authors showed phenotypes evaluated in 2015 and 2016 in Wuhan, the same position. I am not sure whether it is reasonable to state “two environments” here.

2. In Supplementary Fig. 2, “EL (A), ear weight (EW) (B), ear diameter (ED) (C), kernel row number (KRN) (D) of recombinant lines and two parents (NILqknr6 and NILqKNR6) were evaluated using a randomized block design with three replicates in 2015 Wuhan spring (black columns) and 2016 Wuhan spring (gray columns), respectively.” The figure did not show gray columns.

3. In Supplementary Fig. 8, please show spikelet-paired meristem (SPM), spikelet meristem (SM) and floret meristem (FM) using arrows in (C) and (E).

4. Fig. 5D, the graphic symbol is not consistent with the columns.

5. Some important literatures were not cited in the manuscript, about meristem development, “AGO18b negatively regulates determinacy of spikelet meristems on the tassel central spike in maize”, doi: 10.1111/jipb.12596; about the breeding, “Maize biology: From functional genomics to breeding application”, doi: 10.1111/jipb.12819.

6. There are some spelling mistakes in the manuscript.

Reviewer #2 (Remarks to the Author):

Comments for authors

Review for “KERNEL NUMBER PER ROW6 regulates grain yield by phosphorylating an Arf GTPase-activating protein in maize”

In this study, the authors cloned a gene controlling number of kernel (KNR6) in a single row in maize. They further demonstrated that the KNR6 has protein kinase activity and is capable of phosphorylating downstream genes. Moreover, the dominant allele led to the increase of yield in hybrid corn. In addition, they found an insertion of a Harbinger family transposon in the recessive allele and this transposon is not absent from the dominant allele and they consider this transposon is responsible for the difference of variation between the two alleles. Overall I think this is an interesting paper and it is practically useful. However, there are indeed a few issues that need to be clarified before publication.

Major comment

Whether "The TE-PAV Is the Causal Variant for KNR6"?

One of the important conclusions is that "The TE-PAV Is the Causal Variant for KNR6" (p.9, row 3). Of course, the TE-PAV is one of the major genetic changes occurred at this loci, so it possible TE-PAV is the causal variant. However, since there are other mutations in and around this gene, there are alternative possibilities. For example, it is possible other changes, such as the L7F switch, also contribute to the differences. In the extreme case, it is not impossible that the TE-PAV is only a marker.

In the abstract the author stated that the TE-PAV "may function as a transcriptional repressor and/or as a promoter of DNA methylation in the KNR6 upstream region to suppress expression of the gene."

This statement is much more reasonable than "The TE-PAV Is the Causal Variant for KNR6" but it is not guaranteed. I agree, it is possible that it serves as a transcription repressor, based on the result from the luciferase experiment. Nevertheless, I would argue that an insertion upstream of a promoter (in the luciferase experiment) is not really the same as an insertion is present in the intron (in the KNR6 gene). Insertions in introns are not uncommon in plants and they don't always generate detectable effect.

I do suspect the methylation of upstream region of KNR6 is responsible (if not fully) for the reduced expression of this gene in the recessive allele. To test this notion, the authors should do similar association analysis as that in Fig. 3A using SNPs in upstream regions. It is unclear to me why the authors are only showing the analysis in transcribed region.

Having said that, I have problems with TE-PAV as "as a promoter of DNA methylation in the KNR6 upstream region".

p.11 row 22 -23, "In changing trend, the CG and CHG methylation of sequence near the Harbinger-like TE was higher than that far from the Harbinger-like TE (Fig. 3I-J)."

Based on Figure 3i, the sequences immediately flanking the TE-PAV (maybe 2kb on each side?) demonstrate very little methylation and the sequences far away (more than 2 kb) are heavily methylated. This is consistent with previous studies that most DNA transposons or genic transposons do not spread methylation.

So the above statement is largely false. Please correct it. Moreover, I don't know how you could get Figure 3J from Figure 3i. The two portions on the methylation level are not consistent at all. I guess the slide window (1200 bp) is too large to mix everything up in Figure 3j. My suggestion is to use 200 bp slide window and 100 bp step. The curve would not be as smooth but that should be fine.

In addition, given that 85% of the maize genome are transposable elements, it is a little bit surprising that in the 30 kb region in Figure 3 there is only one small Helitron (in addition to the Harbinger element). Please carefully annotate the transposons in this region, particularly the upstream region to see whether there is something readily to be methylated.

So I would like to reiterate my suggestions for this part: 1) conduct an association analysis (I assume you have the data) with SNP in the upstream region of the gene to see whether they are associated with the variation; 2) carefully annotate the transposons in this region; 3) reduce the window size in Figure 3j; 4) turn the tune down about the role of TE-PAV and discuss alternative possibilities.

Minor comments:

1. In the abstract and most places in the text, it was stated the TE-PAV is located in the 5' UTR. However, According to Fig. 1, the insertion is actually in intron. In my opinion, the impact in UTR is very different from that in intron. So please be accurate.

2. Fig. 2 Function of KNR6 on KNR and flowering time.

I did not see anything about flowering time on this Figure. Please double check.

3. Fig. 3A. The 1 kb bar does not look right to me. According to the text, the cDNA is only 1.8 kb in length. According to this bar, it would be around 20 kb. So please make them consistent.

4. Fig. 5D, the legend inside the figure is not consistent with the columns. It should be yellow and green but it is grey and dark right now.

5. Fig. 5G, there should be a letter on top of the first green column.

6. p.18 row 3 to 4. "Sequences from 5'- and 3'- RACE products were assembled to obtain full-length cDNA sequence of KNR6."

I looked at supplemental Fig. 4. It appears that the 5'- RACE product is about 500 bp and 3'- RACE product is about 250 bp. So it is unclear to me how you could get a 1.8 kb full-length cDNA through assembling 500 bp and 250 bp. Please clarify.

In addition, it would help to put the gene structure under this figure and diagram where the gene-specific primers are located in the 5 RACE and 3 RACE experiments.

Reviewer #3 (Remarks to the Author):

The manuscript by Jia et al. reports the cloning and molecular characterization of KRN6, a previously identified kernel row number QTL in maize. Analysis of the QTL in this manuscript is original, thorough and of high quality, leading the authors to identify a transposon insertion in a kinase gene as the causal variant for the KRN6 QTL locus, and two likely in vivo kinase substrates, one of which is an AGAP that when mutated also has a kernel number phenotype in the anticipated direction. Interestingly, the high KRN allele affects meristem size in the inflorescence. Overall the findings are substantiated by an extensive mix of well executed quantitative genetics, molecular genetics and field-based yield trial experiments that document effects of the QTL in inbred breeding and hybrid production genotypes. From these results applications of KNR6 and AGAP are easily envisioned that may increase crop grain yields. The main conclusions are robust and reliable, leading the authors to hypothesize a direct connection of the KRN6 kinase to modulation or auxin levels. This research represents a substantial advancement in our understanding of the developmental and molecular bases of a key agronomic trait, and further investigation such as of the details of effects on auxin pathways, are not necessary. All sections of the manuscript well written and referenced. The work should be of broad interest to researchers in basic and applied plant biology and in related agricultural sciences, such as quantitative genetics and plant breeding.

I have only a few, minor comments for the authors:

p12 line 22 refer the reader to Supp Fig 9, for details of characterizing the mutation in AGAP produced by CRISPR/Cas9, including information about the guide RNAs and methodology.

p13 line 8 Refers to the wrong figure for phosphorylation of 14-3-3 protein by KNR6. Should be Fig. S10 not Fig. 4.

p17 line 20 Refers to the wrong figure for details of CRISPR/Cas9 experiment. Should be Fig. S9, not Fig. S10.

Reviewer #1 (Remarks to the Author):

Comments:

In the manuscript "*KERNEL NUMBER PER ROW6* regulates grain yield by phosphorylating an Arf GTPase-activating protein in maize", the authors established that KERNEL NUMBER PER ROW6 (KNR6) encodes a serine/threonine protein kinase that controls maize grain yield by determining pistillate floret number, ear length and kernel number per row, through fine-mapping, association mapping, expression analysis and transgenic validation. They found a Harbinger-like transposable element (TE) in the 5'-untranslated region of KNR6 is the causal variant to suppress expression of the gene and this TE has a strong impact on grain yield. Further, KNR6 regulates kernel number and grain yield by influencing AGAP phosphorylation to modulate ear inflorescence development. In this manuscript, the authors found a QTL KERNEL NUMBER PER ROW6 (KNR6) control kernel number per row (KNR) and ear length (EL), and elucidated its functional mechanism and the potential in upregulating grain yield. This finding will be of great significance to agricultural breeders. The whole manuscript is well organized and the results are presented with solid evidence and influent English. However, there are still some points need to be improved.

Major points:

1. The authors showed AGAP proteins are phosphorylated by KNR6 by phosphorylation assay in vitro, how about AGAP phosphorylation level in NIL^{qKNR} and NIL^{qknr} plants in vivo?

Response: Thanks for the suggestion. We agree that it is critical to understand the mechanism of how *KNR6* acts in inflorescence meristems, and is a long-term goal of our research. Identification and isolation of this gene is just the beginning of this project. As the reviewer suggested, identifying the phosphorylation substrates of KNR6 in meristems is one of the research tasks. For this, we are breeding the key lines, including NILs and transgenic lines, at our winter maize nursery, in order to collect sufficient meristem samples for phosphoproteomics. We agree our current results are preliminary and do not fully answer the underlying mechanism. Our *in vitro* evidence finds AGAP as a target protein of KNR6 phosphorylation, but we agree *in vivo* evidence is lacking. However we think this is beyond the scope of our current paper, which focuses on the genetics of KNR6. Further evidence on how KNR6 acts in meristems, including from phosphoproteomics, will be presented in an independent paper.

2. It is not suitable to draw out the role of KNR6 on 14-3-3 proteins just based on previous results from Arabidopsis and no evidence in maize. In addition, in the model of Fig. 4J, the hypothetical functions of AGAP and 14-3-3 in “auxin influx” and “physiological adaptation” is exaggerated and need to be modified.

Response: Thanks for the comment, we agree there is no support for this model in maize. We therefore re-drew the putative pathway of *KNR6* and removed the hypothetical functions of AGAP and 14-3-3 in “auxin influx” and “physiological adaptation” from Fig. 4J.

3. The phase “meristematic activity” appeared many times in the manuscript. How to define “meristematic activity”? In Fig.1A, the yellows bars indicated the size or the shape of the inflorescence meristem. But no activity was tested using meristem markers or other methods. I think “meristematic activity” is not accurate here.

Response: Thanks for this comment. We have not used markers to support this claim, and so we replaced the phase "meristematic activity" using appropriate words, such as " stronger ability to produce florets" and " floral production on the ear inflorescence".

4. Fig. 5G, phenotypes of maize grown in Wuhan and Zhengzhou were compared. Grown conditions in these two positions should be described in the text.

Response: Thanks. We described the grown conditions in two locations in the materials and methods section in detail, and also described briefly in the Figure Legends of Figure 5.

5. “Fig. 2 Function of KNR6 on KNR and flowering time.” and “Supplementary Table 2 Phenotypes of ear-related traits and flowering time of transgenic lines.” Why mentioned “flower time”? No results of flower time were showed in these figures.

Response: Thanks for the correction. We removed " flowering time" from in the text.

Minor points:

1. In “Supplementary Fig. 1 Plant and ear status of two parental lines and kernel number per row of the recombinant lines under two environments”, the authors showed phenotypes evaluated in

2015 and 2016 in Wuhan, the same position. I am not sure whether it is reasonable to state “two environments” here.

Response: Thanks. We agree, and deleted the phrase "two environments".

2. In Supplementary Fig. 2, “EL (A), ear weight (EW) (B), ear diameter (ED) (C), kernel row number (KRN) (D) of recombinant lines and two parents (NIL^{qknr6} and NIL^{qKNR6}) were evaluated using a randomized block design with three replicates in 2015 Wuhan spring (black columns) and 2016 Wuhan spring (gray columns), respectively.” The figure did not show gray columns.

Response: Thanks. Phenotype data of the recombinant lines were collected at Wuhan in 2015 and 2016 spring, and were shown in gray and black columns, respectively, in the updated Supplementary Fig. 2.

3. In Supplementary Fig. 8, please show spikelet-paired meristem (SPM), spikelet meristem (SM) and floret meristem (FM) using arrows in (C) and (E).

Response: Thanks for this suggestion. We labeled inflorescence meristem (IM), spikelet-pair meristem (SPM) and spikelet meristem (SM) in (C) and (E) in Supplementary Figure 8.

4. Fig. 5D, the graphic symbol is not consistent with the columns.

Response: Thanks, we corrected this in the updated version.

5. Some important literatures were not cited in the manuscript, about meristem development, “AGO18b negatively regulates determinacy of spikelet meristems on the tassel central spike in maize”, doi: 10.1111/jipb.12596; about the breeding, “Maize biology: From functional genomics to breeding application”, doi: 10.1111/jipb.12819.

Response: Thanks for pointing this out. We added the references about breeding, “Yan J and Tan B-C (2019) Maize biology: From functional genomics to breeding application. J Integr Plant Biol, 61:654-657", and DNA methylation, "Makarevitch I, et al. (2015) Transposable elements contribute to activation of maize genes in response to abiotic stress. PLoS Genetics 11:e1004915" in the Reference list. We didn't cite the article on AGO18b because it is not strongly associated with our story.

6. There are some spelling mistakes in the manuscript.

Response: Thanks. All typographical, grammatical and tense errors were corrected in the updated version.

Reviewer #2 (Remarks to the Author):

Major comment

Whether "The TE-PAV Is the Causal Variant for KNR6"?

One of the important conclusions is that "The TE-PAV Is the Causal Variant for KNR6" (p.9, row 3). Of course, the TE-PAV is one of the major genetic changes occurred at this loci, so it possible TE-PAV is the causal variant. However, since there are other mutations in and around this gene, there are alternative possibilities. For example, it is possible other changes, such as the L7F switch, also contribute to the differences. In the extreme case, it is not impossible that the TE-PAV is only a marker.

Response: Thanks for the suggestion. According the comment, we detected 433 variants including a long terminal repeat (LTR) retrotransposon PAV, the harbinger-like TE PAV and 431 SNPs with minor allele frequencies ≥ 0.05 within 100-kb region centered on *KNR6* in the association panel. Using the 433 variants, we found that a total of 52 variants including the *Harbinger*-like TE-PAV, the LTR-PAV, and other 50 variants were significantly associated with kernel number per row (p=8.81× 10^{-5}). Because of strong linkage disequilibrium, when the *Harbinger*-like TE-PAV was conditioned under an MLM K + Q model, neither LTR-PAV nor other 50 variants were significantly associated with KNR. Similarly, when the LTR-PAV was conditioned, neither *Harbinger*-like TE-PAV nor other 50 variants were significantly associated with KNR. Furthermore, because both TE-PAV and LTR-PAV were two major variants in two parental lines and in the association panel, and were located on *KNR6* regulatory regions that are extremely important for gene expression, thus were referred to as two key genetic markers of the causal variants for *qKNR6*. Using the two markers, two haplotypes were identified in the association panel: Haplotype 1 (*Hap1*) including 48 inbred lines that carry the *Harbinger*-like TE and the LTR, and Haplotype 2 (*Hap2*) including 176 inbred lines that lack the *Harbinger*-like TE and the LTR in *qKNR6*.The average expression level of *KNR6* in the *Hap2* lines was significantly higher than

that in the *Hap1* lines (p = 0.023) (Fig. 3*d*), and the *Hap2* lines had longer ears with more KNR than the *Hap1* lines (Fig. 3*e, f*).

In the abstract the author stated that the TE-PAV "may function as a transcriptional repressor and/or as a promoter of DNA methylation in the KNR6 upstream region to suppress expression of the gene." This statement is much more reasonable than "The TE-PAV Is the Causal Variant for KNR6" but it is not guaranteed. I agree, it is possible that it serves as a transcription repressor, based on the result from the luciferase experiment. Nevertheless, I would argue that an insertion upstream of a promoter (in the luciferase experiment) is not really the same as an insertion is present in the intron (in the KNR6 gene). Insertions in introns are not uncommon in plants and they don't always generate detectable effect.

I do suspect the methylation of upstream region of KNR6 is responsible (if not fully) for the reduced expression of this gene in the recessive allele. To test this notion, the authors should do similar association analysis as that in Fig. 3A using SNPs in upstream regions. It is unclear to me why the authors are only showing the analysis in transcribed region.

Having said that, I have problems with TE-PAV as "as a promoter of DNA methylation in the KNR6 upstream region".

p.11 row 22 -23, "In changing trend, the CG and CHG methylation of sequence near the Harbinger-like TE was higher than that far from the Harbinger-like TE (Fig. 3I-J)."

Based on Figure 3i, the sequences immediately flanking the TE-PAV (maybe 2kb on each side?) demonstrate very little methylation and the sequences far away (more than 2 kb) are heavily methylated. This is consistent with previous studies that most DNA transposons or genic transposons do not spread methylation.

So the above statement is largely false. Please correct it. Moreover, I don't know how you could get Figure 3J from Figure 3i. The two portions on the methylation level are not consistent at all. I guess the slide window (1200 bp) is too large to mix everything up in Figure 3j. My suggestion is to use 200 bp slide window and 100 bp step. The curve would not be as smooth but that should be fine.

In addition, given that 85% of the maize genome are transposable elements, it is a little bit surprising that in the 30 kb region in Figure 3 there is only one small Helitron (in addition to the

Harbinger element). Please carefully annotate the transposons in this region, particularly the upstream region to see whether there is something readily to be methylated.

So I would like to reiterate my suggestions for this part: 1) conduct an association analysis (I assume you have the data) with SNP in the upstream region of the gene to see whether they are associated with the variation; 2) carefully annotate the transposons in this region; 3) reduce the window size in Figure 3j; 4) turn the tune down about the role of TE-PAV and discuss alternative possibilities.

Response:

Response: Thanks very much for the suggestions that are of great help to improve this article. As suggested, we first carefully annotated the transposons in the flanking regions of KNR6, including three LTR retrotransposons and a Helitron, and found an LTR retrotransposon PAV approximately 5.1-kb upstream of the *KNR6* transcription start site in the association panel and between the two parental lines. These updated results are described in Page7 Line#16-20.

Second, we identified 433 variants within a 100-kb region centered on *KNR6*, and performed association analysis and conditional association analysis. We found, in addition to the Harbinger-like TE PAV, the LTR-PAV was also associated with Kernel number per row (KNR). We also analyzed the genetic effects of Haplotypes on KNR and EL (ear length). These updated results are described in the text. Third, we re-analyzed methylation levels in three contexts within an approximately 30-kb genomic region centered on *KNR6* in both parents using a window size of 200 bp and a step size of 100 bp, these updated results are shown in Fig.3*j* and *k*, and Page11 Line13-25. We found, in NIL^{qknr6}, both the LTR-PAV and the TE-PAV were hypermethylated in CG (94.5% and 97.1%), and CHG (85.2% and 63.4%) contexts, but not in CHH (1.7% and 7.0%) (Fig. 3*j*). Moreover, the average methylation level in the 5 - 15-kb region upstream from the *Harbinger*-like TE was dramatically higher in NIL^{qknr6} than in NIL^{qKNR6} (Fig. 3*i*, *k*), while the regions immediately flanking the TE-PAV had low methylation (Fig. 3*i*,*k*). This result agrees with the observation that genic transposons do not always spread methylation (Makarevitch I, et al. Transposable elements contribute to activation of maize genes in response to abiotic stress. *PLoS Genetics,* 2015, 11:e1004915). The heavily methylated regions 5 - 15-kb upstream region from the *Harbinger*-like TE correspond to the LTR-PAV, suggesting methylation difference at the distal upstream region of *KNR6* between the two parent lines may not be caused by the *Harbinger*-like

TE-PAV but by the LTR-PAV, which is located 5.1-kb upstream of *KNR6*. In addition, a high methylation level was also found within the 5 kb region downstream of *KNR6* in both parents, due to the presence of a *Helitron* TE and an LTR retrotransposon (Fig. 3*i-j*).

Finally, we rewrote the paragraph on DNA methylation.

Minor comments:

1. In the abstract and most places in the text, it was stated the TE-PAV is located in the 5' UTR. However, According to Fig. 1, the insertion is actually in intron. In my opinion, the impact in UTR is very different from that in intron. So please be accurate.

Response: Thanks. The phrase "5'-UTR "is replaced by " an intron in the 5-UTR" throughout.

2. Fig. 2 Function of KNR6 on KNR and flowering time.

I did not see anything about flowering time on this Figure. Please double check.

Response: thanks for pointing out this error, we deleted "flowering time" in the title of Fig. 2.

3. Fig. 3A. The 1 kb bar does not look right to me. According to the text, the cDNA is only 1.8 kb in length. According to this bar, it would be around 20 kb. So please make them consistent.

Response: Thanks for pointing this out, we corrected this bar in Fig.3a.

4. Fig. 5D, the legend inside the figure is not consistent with the columns. It should be yellow and green but it is grey and dark right now.

Response: Thanks for the suggestion. We correct the legends in Fig.5d.

5. Fig. 5G, there should be a letter on top of the first green column.

Response: Thanks. We correct the legends in Fig.5g.

6. p.18 row 3 to 4. "Sequences from 5′- and 3′- RACE products were assembled to obtain full-length cDNA sequence of KNR6." I looked at supplemental Fig. 4. It appears that the 5′-RACE product is about 500 bp and 3′- RACE product is about 250 bp. So it is unclear to me how you could get a 1.8 kb full-length cDNA through assembling 500 bp and 250 bp. Please clarify. In addition, it would help to put the gene structure under this figure and diagram where the gene-specific primers are located in the 5 RACE and 3 RACE experiments.

Response: Thanks. We agree this was not clear, and added the gene structure graphic (Supplementary Fig. 4*a*) and marked out the location of primers on the gene. We provided a gel electrophoresis map of full-length cDNA amplification (Supplementary Fig. 4*d*). The updated Supplementary Fig. 4 is shown as follows.

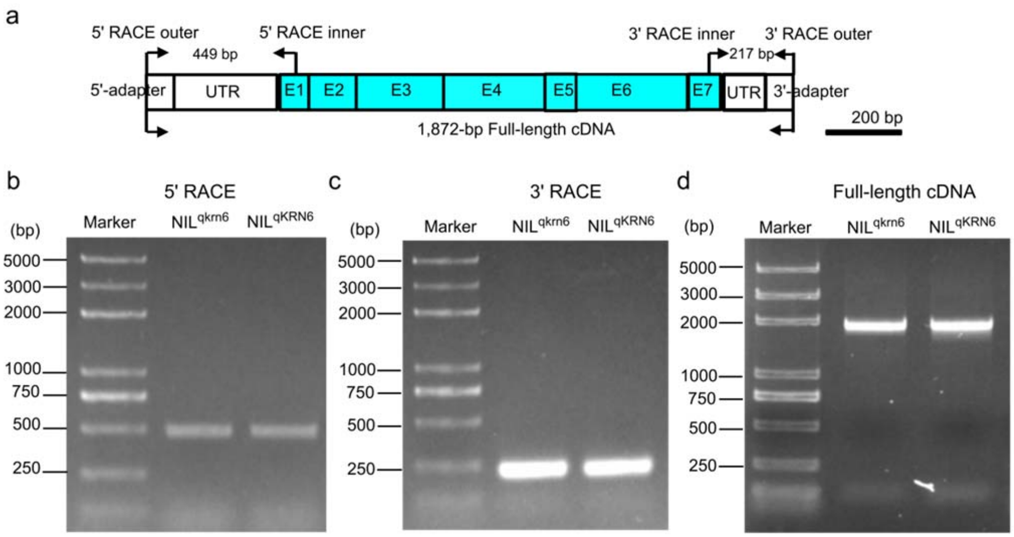

Supplementary Fig. 4 Rapid amplification of *KNR6* cDNA ends in NIL^{qknr6} and NIL^{qKNR6}.

(***a***) A graphical representation of positions of primers for Rapid amplification of *KNR6* cDNA ends. E: Exon. UTR: un-translational region. (***b***) The gel electrophoresis results of 5′-RACE of *KNR6* in the NIL^{qknr6} and NIL^{qKNR6}. (***c***) The gel electrophoresis results of 3'-RACE of *KNR6* in the NIL^{qknr6} and NIL^{qKNR6}. (***d***) The gel electrophoresis results of full-length cDNA amplification of *KNR6* in the NIL^{qknr6} and NIL^{qKNR6}, respectively.

Reviewer #3 (Remarks to the Author):

The manuscript by Jia et al. reports the cloning and molecular characterization of KRN6, a previously identified kernel row number QTL in maize. Analysis of the QTL in this manuscript is original, thorough and of high quality, leading the authors to identify a transposon insertion in a kinase gene as the causal variant for the KRN6 QTL locus, and two likely in vivo kinase substrates, one of which is an AGAP that when mutated also has a kernel number phenotype in the anticipated direction. Interestingly, the high KRN allele affects meristem size in the inflorescence. Overall the findings are substantiated by an extensive mix of well executed quantitative genetics, molecular genetics and field-based yield trial experiments that document effects of the QTL in inbred breeding and hybrid production genotypes. From these results applications of KNR6 and AGAP are easily envisioned that may increase crop grain yields. The main conclusions are robust and reliable, leading the authors to hypothesize a direct connection of the KRN6 kinase to modulation or auxin levels. This research represents a substantial advancement in our

understanding of the developmental and molecular bases of a key agronomic trait, and further investigation such as of the details of effects on auxin pathways, are not necessary. All sections of the manuscript well written and referenced. The work should be of broad interest to researchers in basic and applied plant biology and in related agricultural sciences, such as quantitative genetics and plant breeding.

I have only a few, minor comments for the authors:

p12 line 22 refer the reader to Supp Fig 9, for details of characterizing the mutation in AGAP produced by CRISPR/Cas9, including information about the guide RNAs and methodology.

p13 line 8 Refers to the wrong figure for phosphorylation of 14-3-3 protein by KNR6. Should be Fig. S10 not Fig. 4.

p17 line 20 Refers to the wrong figure for details of CRISPR/Cas9 experiment. Should be Fig. S9, not Fig. S10.

Response: Sorry for the confusion. All typos, grammatical and tense errors are corrected in the updated version.

REVIEWERS' COMMENTS:

Reviewer #1 (Remarks to the Author):

Comments:

In this manuscript, the authors found a QTL KERNEL NUMBER PER ROW6 (KNR6) control kernel number per row (KNR) and ear length (EL), and elucidated its functional mechanism and the potential in upregulating grain yield. The second version of the manuscript was much improved. However, there is still one point not satisfied.

Major points:

1. The title "KERNEL NUMBER PER ROW6 regulates grain yield by phosphorylating an Arf GTPase-activating protein in maize" contains two points, the first one KNR regulates grain yield was elucidated by fine-mapping, association mapping, expression analysis and transgenic validation in the manuscript. The second one described the functional mechanism of KNR by phosphorylating AGAP. In this part, the authors picked up AGAP through IP-MS and provided evidence that KNR phosphorylated AGAP as a kinase in vitro. More importantly, the Crispr line of AGAP showed similar phenotype in ear length and yield. However, there is a small logic gap in this part. Direct evidence that indicates the phenotype of AGAP Crispr line was caused by KNR phosphorylation was missed. At this stage, the authors couldn't exclude the possibilities that other factors such as other kinases also regulate ear length through AGAP. Based on the response to my first question in the last version, it seems difficult to provide in vivo phosphorylation results, so I suggest the authors to modify the title to attenuate the second part.

Minor points:

1. Supplemental Fig8 C and E may move to the main text.

Reviewer #2 (Remarks to the Author):

Review for "KERNEL NUMBER PER ROW6 regulates grain yield by phosphorylating an Arf GTPase-activating protein in maize".

This is a revision. I appreciate the effort from the author to generate and analyze the new data, which significantly improved the manuscript. I only have some minor comments:

1. Row 70 “functional variation with wide significance.”

“wide significance” sounds odd. How about “functional variation with profound significance.”?

2. Row 125-126 “the two QTL parents produced an identical 1,872_bp KNR6 transcript that encoded a protein of 381 amino acids”

If I understand it correctly, there are several SNPs within the cDNA, including the non-synonymous mutations. As a result, I don’t think you could say they are identical. So maybe change to “the two QTL parents produced transcripts with identical length (1,872 bp), and they encoded proteins of 381 amino acids”.

3. Row 233 – 235 “suggesting methylation differences in the upstream region of KNR6 between the two parent lines may be caused not by the Harbinger-like TE-PAV, but by the LTR-PAV 5.1_kb upstream of KNR6.”

I think the sentence should be reworded to “suggesting methylation differences in the upstream region of KNR6 between the two parent lines may be caused by the LTR-PAV 5.1_kb upstream of KNR6, not by the Harbinger-like TE-PAV within the first intron of KNR6.”

4. Row 305 – 306 “Compared with hybrids resulting from crosses with NILqKNR6, the EL of hybrids crossed from NILqKNR6 increased…”

I believe the first “qKNR6” should be “qknr6”, please correct it.

Reviewer #3 (Remarks to the Author):

For the authors: My comments on the previous version of the manuscript were brief and minor, and have been addressed in the revision. I find the revised manuscript only improved, thanks in part to some excellent comments by the other two reviewers.

Reviewer 1's major point #1 relates to in vivo confirmation of phosphorylation of AGAP by KNR6. In my opinion, while that unanswered question is certainly compelling, those in vivo data are beyond the scope of the current manuscript. The import and impact of the manuscript stands alone without that in vivo mechanistic data, in substantially advancing understanding of the developmental and molecular bases of a key agronomic trait.

I did see a typo: Supp Figure 4, legend: "UTR: un-translational region" should say "UTR: un-translated region"

Response to reviewer's comments:

Reviewer #1 (Remarks to the Author):

In this manuscript, the authors found a QTL KERNEL NUMBER PER ROW6 (KNR6) control kernel number per row (KNR) and ear length (EL), and elucidated its functional mechanism and the potential in upregulating grain yield. The second version of the manuscript was much improved. However, there is still one point not satisfied.

Major points:

Q1. The title "KERNEL NUMBER PER ROW6 regulates grain yield by phosphorylating an Arf GTPase-activating protein in maize" contains two points, the first one KNR regulates grain yield was elucidated by fine-mapping, association mapping, expression analysis and transgenic validation in the manuscript. The second one described the functional mechanism of KNR by phosphorylating AGAP. In this part, the authors picked up AGAP through IP-MS and provided evidence that KNR phosphorylated AGAP as a kinase in vitro. More importantly, the Crispr line of AGAP showed similar phenotype in ear length and yield. However, there is a small logic gap in this part. Direct evidence that indicates the phenotype of AGAP Crispr line was caused by KNR phosphorylation was missed. At this stage, the authors couldn't exclude the possibilities that other factors such as other kinases also regulate ear length through AGAP. Based on the response to my first question in the last version, it seems difficult to provide in vivo phosphorylation results, so I suggest the authors to modify the title to attenuate the second part.

Response: Thanks. We fully agree with the critical comment. Since it is difficult to us to provide in vivo evidence in a short period of time, we chose to revise the title to attenuate the KNR phosphorylation to AGAP. The revised title is "*KERNEL NUMBER PER ROW6* encoding a serine/threonine protein kinase regulates maize grain yield".

Minor points:

Q2. Supplemental Fig8 C and E may move to the main text.

Response: Thanks. The Supplemental Fig. 8 c and 8e have been moved to the main text, and have been integrated into Figure 4.

Reviewer #2 (Remarks to the Author):

This is a revision. I appreciate the effort from the author to generate and analyze the new data, which significantly improved the manuscript. I only have some minor comments:

1. Row 70 “functional variation with wide significance.”

“wide significance” sounds odd. How about “functional variation with profound significance.”?

Response: Thanks. We revised it according the comment.

2. Row 125-126 “the two QTL parents produced an identical 1,872_bp KNR6 transcript that encoded a protein of 381 amino acids”. If I understand it correctly, there are several SNPs within the cDNA, including the non-synonymous mutations. As a result, I don’t think you could say they are identical. So maybe change to “the two QTL parents produced transcripts with identical length (1,872 bp), and they encoded proteins of 381 amino acids”.

Response: Thanks. We revised it according the comment.

3. Row 233 – 235 “suggesting methylation differences in the upstream region of KNR6 between the two parent lines may be caused not by the Harbinger-like TE-PAV, but by the LTR-PAV 5.1_kb upstream of KNR6.” I think the sentence should be reworded to “suggesting methylation differences in the upstream region of KNR6 between the two parent lines may be caused by the LTR-PAV 5.1_kb upstream of KNR6, not by the Harbinger-like TE-PAV within the first intron of KNR6.”

Response: Thanks. We rephrased the sentence according to the comment.

4. Row 305 – 306 “Compared with hybrids resulting from crosses with NIL^{qKNR6}, the EL of hybrids crossed from NIL^{qKNR6} increased…” I believe the first “qKNR6” should be “qknr6”, please correct it.

Response: I apologize for this typo. We corrected it in the updated version.

Reviewer #3 (Remarks to the Author):

For the authors: My comments on the previous version of the manuscript were brief and minor, and have been addressed in the revision. I find the revised manuscript only improved, thanks in part to some excellent comments by the other two reviewers.

Reviewer 1's major point #1 relates to in vivo confirmation of phosphorylation of AGAP by KNR6. In my opinion, while that unanswered question is certainly compelling, those in vivo data are beyond the scope of

the current manuscript. The import and impact of the manuscript stands alone without that in vivo mechanistic data, in substantially advancing understanding of the developmental and molecular bases of a key agronomic trait. I did see a typo: Supp Figure 4, legend: "UTR: un-translational region" should say "UTR: un-translated region"

Response: I apologize for this typo. We corrected it in the updated version.
